# Spatio-temporal patterns in floral resources and plant-pollinator network structure in the Alaskan Arctic

**DOI:** 10.3389/fpls.2025.1552422

**Published:** 2025-09-24

**Authors:** Roxaneh S. Khorsand, Zachary R. Ginn, Flavia Sancier-Barbosa

**Affiliations:** ^1^ Department of Organismal Biology and Ecology, Colorado College, Colorado Springs, CO, United States; ^2^ Department of Mathematics and Computer Science, Colorado College, Colorado Springs, CO, United States

**Keywords:** Low Arctic, floral resources, plant-visitor networks, phenology, pollination, tundra

## Abstract

Predicting shifts in plant-pollinator communities as a result of warming requires an accurate understanding of floral availability, insect activity, and spatio-temporal patterns of plant-insect interaction. Plant-insect visitor network studies from the High Arctic have demonstrated high generalization and rapid temporal turnover, yet comparable data are lacking for the Low Arctic. We worked in two tundra plant community types on the North Slope of Alaska in 2022 and 2023 to construct the first plant-insect visitor networks for this region of the Arctic and document temporal patterns of floral resource availability and insect visitation. We found temporal differences in floral availability between community types. Both floral density and the number of species in anthesis peaked earlier in the dry heath tundra compared to the moist acidic tundra. In addition, Hymenopteran visitation rates showed a bimodal peak (early- and late-season) while Dipteran visitation rates showed a unimodal pattern. Network complexity peaked earlier in the dry compared to the moist community. Our results suggest that temporal heterogeneity in floral resources between plant community types may increase the duration of floral availability for insects at a landscape scale. Given this region’s low species diversity and increasing vulnerability to extreme weather events, spatio-temporal heterogeneity in floral resources may play a critical role in the resiliency of this system.

## 1 Introduction

The Arctic is warming up to four times the rate of the global average as a result of Arctic amplification (Rantanen et al., 2022). Warming may impact biotic communities at multiple levels, including mutualistic interactions between tundra plants and pollinators. Plant biotic responses to warming have been well documented, specifically shifts in community composition and variation in phenology. For example, erect, deciduous shrubs are becoming increasingly dominant in Arctic plant communities (Myers-Smith et al., 2011; Elmendorf et al., 2012; Henry et al., 2022). Shrubification may result in decreased competitive ability of prostrate species, many of which provide critical floral rewards and berries to insects, birds, and mammals (Arft et al., 1999; Ikeda et al., 2015; Kettenbach et al., 2017). In addition to compositional changes of the Arctic tundra, phenological shifts including advanced flowering are well documented (Chapin and Shaver, 1996; Wipf and Rixen, 2010; Oberbauer et al., 2013; Bjorkman et al., 2015; Khorsand Rosa et al., 2015; Semenchuk et al., 2016; Collins et al., 2021). The duration of the flowering season appears to be increasing at the community level (Collins et al., 2021) although other studies show contraction of flowering duration because of earlier onset by late-flowering species (Høye et al., 2013; Prevéy et al., 2019). These contrasting results justify further research on the duration of floral resource availability across tundra plant communities, as well as within specific community types.

Plant-pollinator interactions represent a dynamic relationship between the abiotic and biotic environment. The Arctic tundra is a patchwork of vegetation types and microsites determined by variation in snow regime including snow accumulation and timing of snowmelt (Molau, 1993; Stanton et al., 1994; Walker, 2000). Snowmelt date has been shown to relate closely to flowering phenology (Billings and Mooney, 1968; Bjorkman et al., 2015). In addition, warming can influence the timing of peak flowering at the community level as well as the overlap of peak flowering among species, with implications for the pollinator community (Gillespie et al., 2016). From the insect perspective, timing of snowmelt appears to be the primary predictor of Arctic insect phenology (Høye and Forchhammer, 2008) and insects further rely on floral resources throughout the growing season (Høye et al., 2007; Kudo and Cooper, 2019). While asexual reproduction and autogamy are considered common in Arctic plants (Molau, 1993), many plant species still depend on insects for optimal seed set (Williams and Batzli, 1982; Philipp et al., 1996; Fulkerson et al., 2012; Tiusanen et al., 2016; Urbanowicz et al., 2018; Koch et al., 2020). Thus, unpacking the complex relationship between floral resources, insect population dynamics, and plant reproductive success in the Arctic is increasingly important in the context of rapid warming.

Bipartite network analyses are critical analytical tools used to describe community-level plant-insect interactions (Jordano, 1987; Olesen and Jordano, 2002). Interactions or links between plants and insects can be represented as a binary (presence/absence) or as a weighted value relating to the strength of interaction (e.g. number of visits observed). Standardized network parameters like nestedness (Bascompte et al., 2003), modularity (Olesen et al., 2007), and connectance (Blüthgen et al., 2006) not only provide a framework for understanding network assembly and stability over time (Gillespie and Cooper, 2022), but also provide a systematic way to describe temporal patterns of plant-visitor interactions (Pradal et al., 2009; Burkle and Alarcón, 2011). A comprehensive understanding of spatio-temporal patterns in plant-visitor networks requires examining these network parameters at a “static” level (using accumulated data from the entire growing season) as well as a “dynamic” level (using data from various “time-slices” or sampling points to determine temporal changes) (Olesen et al., 2008; Pradal et al., 2009).

Temporal dynamics of plant-visitor networks have been shown to vary within a single growing season. For example, in the High Arctic, Olesen et al. (2008) found that network dynamics were stable across years but varied substantially within a single growing season. Similarly, Cirtwill et al. (2023) reported more within-year-variation in the High Arctic network structure than between-year-variation. This variation may be linked to strong seasonal changes characteristic of the Arctic tundra. In addition to the inherently stressful and highly variable abiotic environment (Kankaanpää et al., 2018), stringent climate conditions impose a short window of time for plants and insects to interact (Pradal et al., 2009; Schmidt et al., 2016). Rapid warming may exacerbate these harsh conditions by increasing the frequency of extreme climactic events and advancing snowmelt, potentially decreasing sexual reproductive success in plants (Panchen et al., 2021) and increasing network specialization (although elevation may also play a role, see Hoiss et al., 2015). Given the Arctic tundra’s strong seasonality, patchy distribution of floral resources, and species-specific flowering phenology, it is crucial to assess the temporal dynamics of the plant-visitor community within a single growing season.

A major concern with accelerated warming and advanced flowering phenology is the potential for plant-pollinator phenological mismatch (Gérard et al., 2020), although evidence of mismatch is still scarce (Iler et al., 2013; Forrest, 2015; Gillespie and Cooper, 2022). Arctic plant-pollinator communities may be particularly vulnerable to asynchrony given the short growing season and low species diversity (Høye et al., 2013; Vasiliev and Greenwood, 2021). Many Arctic plant species have brief flowering phenophases of only a few weeks leading to rapid turnover of species within the growing season (Olesen et al., 2008; Cirtwill et al., 2018). Furthermore, Arctic networks have been shown to exhibit strong temporal dynamics (Pradal et al., 2009; Semenchuk et al., 2016; Gillespie and Cooper, 2022). Although floral resources in this harsh environment are relatively limited, a generalist network, characterized by high generalization and flexible resource use by insects (Steffan-Dewenter et al., 2006), can increase the number of potential plant-pollinator interactions and potentially buffer individual species from phenological mismatch (Gillespie and Cooper, 2022). Thus, determining the degree of network generalization is a crucial step in accurately predicting the resiliency of the plant-pollinator community (Burkle et al., 2013; Caradonna et al., 2017).

We investigated the spatio-temporal dynamics of floral resources, insect visitation, and network structure over two growing seasons in two tundra plant community types, dry heath and moist acidic, on the North Slope of Alaska. Specifically, we observed floral phenology and collected insect floral visitors to construct static and dynamic plant-visitor networks. Our study addresses the following research questions: (1) How do floral resources, defined as floral density and number of species in anthesis, vary spatially and temporally throughout the growing season? (2) How does insect visitation vary spatially and temporally throughout the growing season? And (3) How does network size and structure change over the growing season as well as across plant community types? To our knowledge, these are the first plant-visitor networks created for this understudied region of the Arctic. These baseline data are crucial to test for potential plant-pollinator phenological mismatch and species compositional shifts as the North Slope continues to warm.

## 2 Materials and methods

### 2.1 Study site and experimental design

We conducted fieldwork in 2022 and 2023 at two sites on the North Slope of Alaska, USA: Toolik Lake (68° 38’ N, 149° 36’ W, elevation 730 m) and Imnavait Creek (68° 37’ N, 148° 18’ W, elevation 930 m), approximately 12 km from each other. The two sites are very similar in terms of climate and timing of snowmelt, although inter-annual variation occurs. Within each site, we worked in two tundra plant community types, dry heath tundra (“Dry”) and moist acidic tundra (“Moist”). Dry heath tundra is generally characterized by shallow snow accumulation and low soil organic matter, high wind exposure, and relatively low vascular plant diversity. In contrast, snowbeds and relatively high soil organic matter, lower wind exposure and higher plant diversity characterize moist acidic tundra. See Walker et al. (1994) and Khorsand et al. (2024) for a description of the field sites and their plant-pollinator communities.

Between June 1 and August 5 of each study year, we quantified flowering phenology, floral density, and insect visitation rates in a total of 64 1m^2^ plots in dry and moist communities (n = 16 plots per community type per site). In addition, we systematically collected insects in two large sampling areas at Toolik only (see section 2.4).

### 2.2 Flowering phenology

At each site, we conducted biweekly phenological surveys on all control plots. During these surveys, we noted all plant species in anthesis in each plot. We defined anthesis (synonymous with “in bloom” or “flowering”) as petals and reproductive structures being intact/not withered, and pollen dispersing from the anthers. Additionally, we quantified floral density in a 30.5 by 30.5 cm frame in the center of each plot by counting all open flowers. For most species, every flower was counted individually. However, we considered an inflorescence as the unit of measurement in a subset of species: *Bistorta officinalis*, all catkin-bearing species including *Salix* spp. and *Betula nana*, and all species of Asteraceae including *Antennaria monocephala* and *Petasites frigidus*.

### 2.3 Floral visitor observations

We performed biweekly, ten-minute floral visitor observations to quantify insect visitation rates in each community at each site (2022: N = 341 observations; 2023: N = 350). Plots were randomly chosen each day from a pool of flowering plots using a digital random number generator. In both years, individual plots were observed no more than three times per day between 09:00 and 17:00 on days with favorable weather conditions (free of rain, major wind, or freezing temperatures). Additional unmarked plots were observed when marked control plots lacked flowers. We defined visitation as an insect landing on a flower and/or contacting floral reproductive structures (anthers or stigmas). For each visitation event, we recorded the following: plant species, visitor insect order, number of visitor individuals, number of distinct visits made, and visitor behavior including foraging activity and duration of visit. Insect collection was not performed during floral visitor observations so as not to affect visitation rates.

### 2.4 Insect collection and identification

We collected insects at least twice a week between 9:00 and 17:00 on days with favorable weather conditions although sampling protocols differed between years. In 2022, insects were collected opportunistically from plots in both community types at both sites. In 2023, we restricted insect collection to both communities only at Toolik and implemented a standardized collection protocol. Standardizing our collection effort on each flowering plant species ensured abundant and rare plant species were sampled equally (Olesen et al., 2008; Gillespie and Cooper, 2022). Other studies have validated this approach to reduce bias toward abundant taxa and accurately reflect network diversity (Gibson et al., 2010; Jordano, 2016). We systematically collected insects in two large sampling areas, one in Dry (2.75 Ha) and one in Moist (2.60 Ha). These sampling areas were established directly adjacent to our plots (where 2022 collections took place). On each collecting day, observers walked a standardized route through each sampling area, noting every plant species in flower and marking rare species with flags. Each plant species was observed in a patch size of up to 2m^2^ for 20 minutes. During each 20-minute period, we netted all flower-visiting insects on that particular plant species. In cases where an insect escaped netting, we identified it to the highest taxonomic level possible and recorded as one observation.

In both years, insects were transferred to clean vials, placed in kill jars charged with ethyl acetate, frozen for 24 hours, then thawed and pinned (Kearns and Inouye, 1993). We identified all insects to order and family, and when possible, species. That said, we were unable to identify all individuals to species as species-level keys were unavailable for some taxa in this region of the Arctic. Therefore, we constructed networks at the family level. We emphasize that our results reflect sampling at the plant species-insect family level and should not be compared to species-species level network studies. While less rigorous than a species-level network, sampling at the insect family level still demonstrates community patterns of interaction and facilitates comparison with other Arctic network studies (Gillespie and Cooper, 2022). Insect voucher specimens are currently being stored in the Entomology Collection at Colorado College with the goal of returning all specimens to Alaska (Museum of the North, UAF).

### 2.5 Statistical analyses

All statistical analyses were performed using R (R Core Team, 2023). The mgcv package (Wood, 2017) was used to fit Generalized Additive Mixed Models (GAMMs), the bipartite package (Dormann et al., 2008) to construct plant-insect interaction networks and run null models, and the vegan package (Oksanen et al., 2024) to evaluate network sampling completeness.

#### 2.5.1 Temporal dynamics with GAM models

To investigate the temporal patterns in the number of species in anthesis, floral density, and insect visitation, we fit GAMMs with a Poisson distribution for the number of species in anthesis and a Tweedie distribution for floral density and insect visitation, which better accounts for over-dispersion and high occurrence of zeroes. When looking at individual species, we modeled the proportion of plots specifically in anthesis out of all plots in which that species occurred (flowering or not) using a GAMM with a binomial distribution. Plant species which received no insect visits were excluded from all GAMMS. The models used penalized thin plate regression spline smoothers with Julian Day as the primary predictor. For species in anthesis and floral density, we modeled the difference in temporal trends between Dry and Moist for each site, with Dry as the reference level. GAMM outputs also provided mean differences between community types for each response variable including the number of species in anthesis. For insect visitation, we modeled the difference between the number of visits per observation period by insect order (Hymenoptera and Diptera) for each of the two community types, with Diptera as the reference level. We also computed models combining communities and sites. In all models, plot identifier was considered a random effect, and estimates were done via restricted maximum likelihood (REML). We visualized predictions with 90% confidence bands to identify weeks in which temporal patterns differed.

#### 2.5.2 Bipartite network analysis

To construct networks, we used insect collections and visitor observations to identify links between plant species and insect families. Data were aggregated into data-matrices based on year, community type, and week, then plotted as networks using the function visweb in bipartite. We define the following terms used to describe our networks: (1) cumulative: both community types; (2) subset: dry or moist community; (3) static: entire growing season; (4) dynamic: growing season separated into weekly time slices; (5) binary: describes the presence/absence of a plant species-insect family link; and (6) weighted: accounts for the frequency of each plant species-insect family link.

We also calculated six network metrices using the function networklevel: Connectance (C), Nestedness (N), Nestedness based on Overlap and Decreasing Fill (NODF), Network-level specialization (H_2_’), Mean number of links per plant species-insect family ( $\bar{L}$ ), and Quantitative modularity (Q). We compared observed static networks to baseline or random networks using bipartite’s nullmodel function with 1000 repetitions and the “r2dtable” method (Dormann et al., 2008). We reported Z scores as a measure of effect size, with positive values indicating that the observed metric was higher than the mean of the simulated values.

C (range 0-1) is defined as the proportion of the actually observed interactions to all possible interactions (Blüthgen et al., 2006). N (0-100) is the extent to which generalist species interact with specialists, and vice versa. A low N value indicates a highly nested network, or a non-random structural pattern generating asymmetrical interactions in which specialist species interact with a subset of partners that interact with the more generalist taxa (Bascompte et al., 2003). NODF (0-100) is a quantitative metric of nestedness that expands beyond binary matrices (presence/absence) and accounts for paired overlap and decreasing fill among columns and rows of the network matrix (Almeida-Neto et al., 2008). H_2_’ (0-1) refers to the degree of specialization in the entire network where 0 indicates extreme generalization and 1 indicates extreme specialization (Blüthgen et al., 2006). $\bar{L}$ is the mean number of links, or interactions, between a plant species and floral visitor family. Q (-1-1) is a measure of how well species interactions or links are organized into modules or functional units within the bipartite network (Paine, 1980; Olesen et al., 2007; Dormann and Strauss, 2014).

For 2022, we constructed a cumulative static network to visualize binary links between plant species and insect families for the entire study area (both Toolik and Imnavait) over the complete growing season. This preliminary network serves to document plant and insect diversity over a larger spatial area on the North Slope. However, owing to the non-standardized collection protocol in 2022 we did not investigate temporal or spatial differences in the 2022 network or compute weighted indices (H_2_’, Q). For 2023, we constructed weighted static networks for each community type (Dry and Moist) at Toolik, as well as a weighted static network for both community types together (cumulative). To investigate patterns in network complexity over the growing season, we also created dynamic networks using weekly time slices for cumulative, dry, and moist datasets. Week one started on May 29, 2023 (DOY 149).

#### 2.5.3 Sampling estimates

The degree to which a community is sampled can influence network structure (Schwarz et al., 2020). We used the Chao1 estimator of asymptotic richness to estimate sampling completeness (Chao, 1984) and sampling coverage (Chao and Jost, 2012) of plants, floral visitors, and links in each network. Sampling completeness refers to the proportion of detected species, families, and links compared to Chao1 estimates (Schwarz et al., 2020). Sampling coverage is a weighted measure of sampling completeness and refers to the proportion of all individuals or interaction events in the community belonging to the species or links represented in the sample used to construct a network (Chao and Jost, 2012). We calculated these values for the static and dynamic networks, obtaining absolute coverage values for the entire growing season, as well as median coverage values considering weekly time slices.

## 3 Results

### 3.1 Spatio-temporal patterns of floral resources

**Figure 1:**
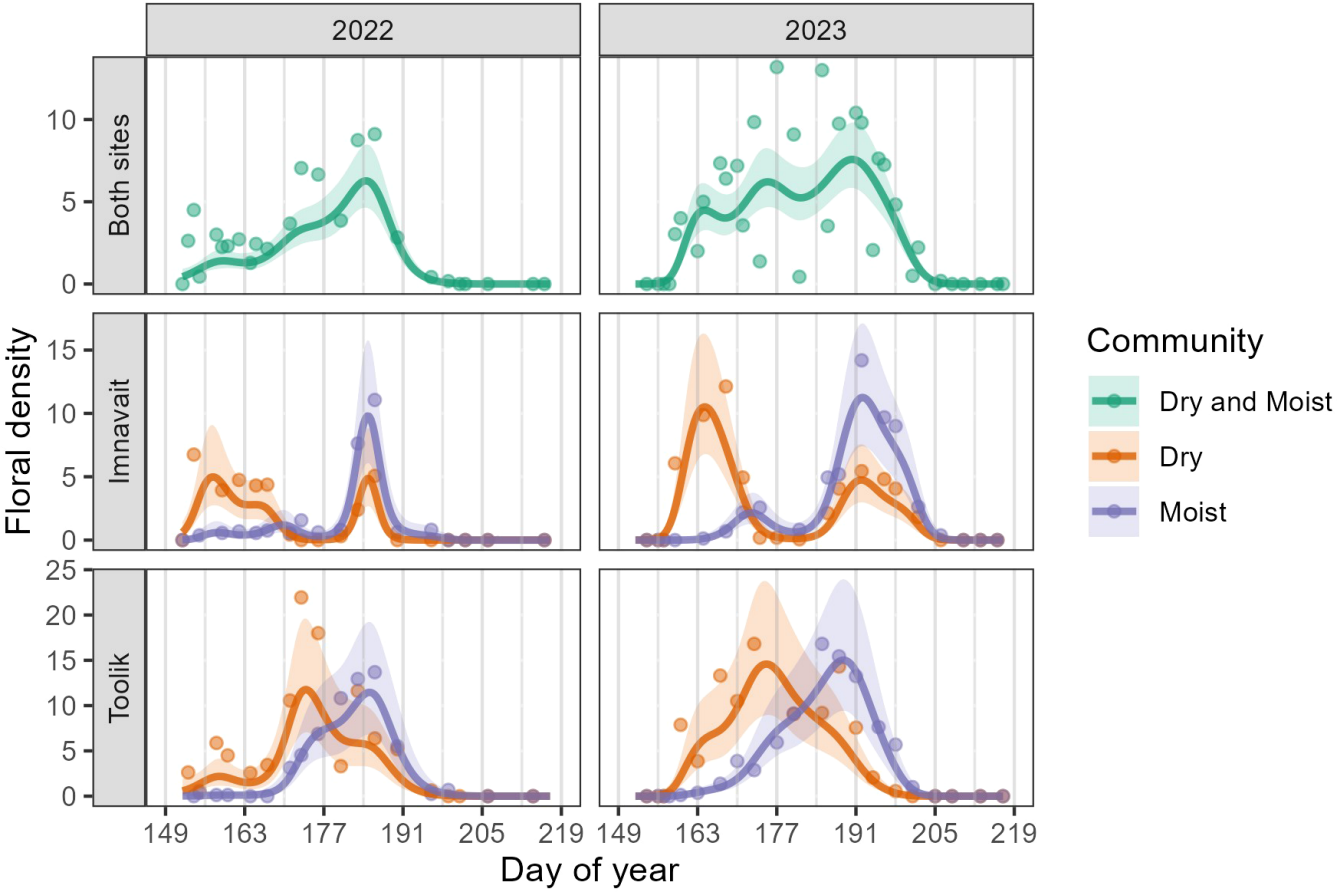
GAMM predictions (smooth trendlines) with 90% confidence bands (shaded) and daily means (points) for floral density per plot. The first row shows Imnavait and Toolik sites combined, while the second and third rows show the individual sites, separated by community type. Gray vertical lines mark the beginning of each study week.

**Figure 2:**
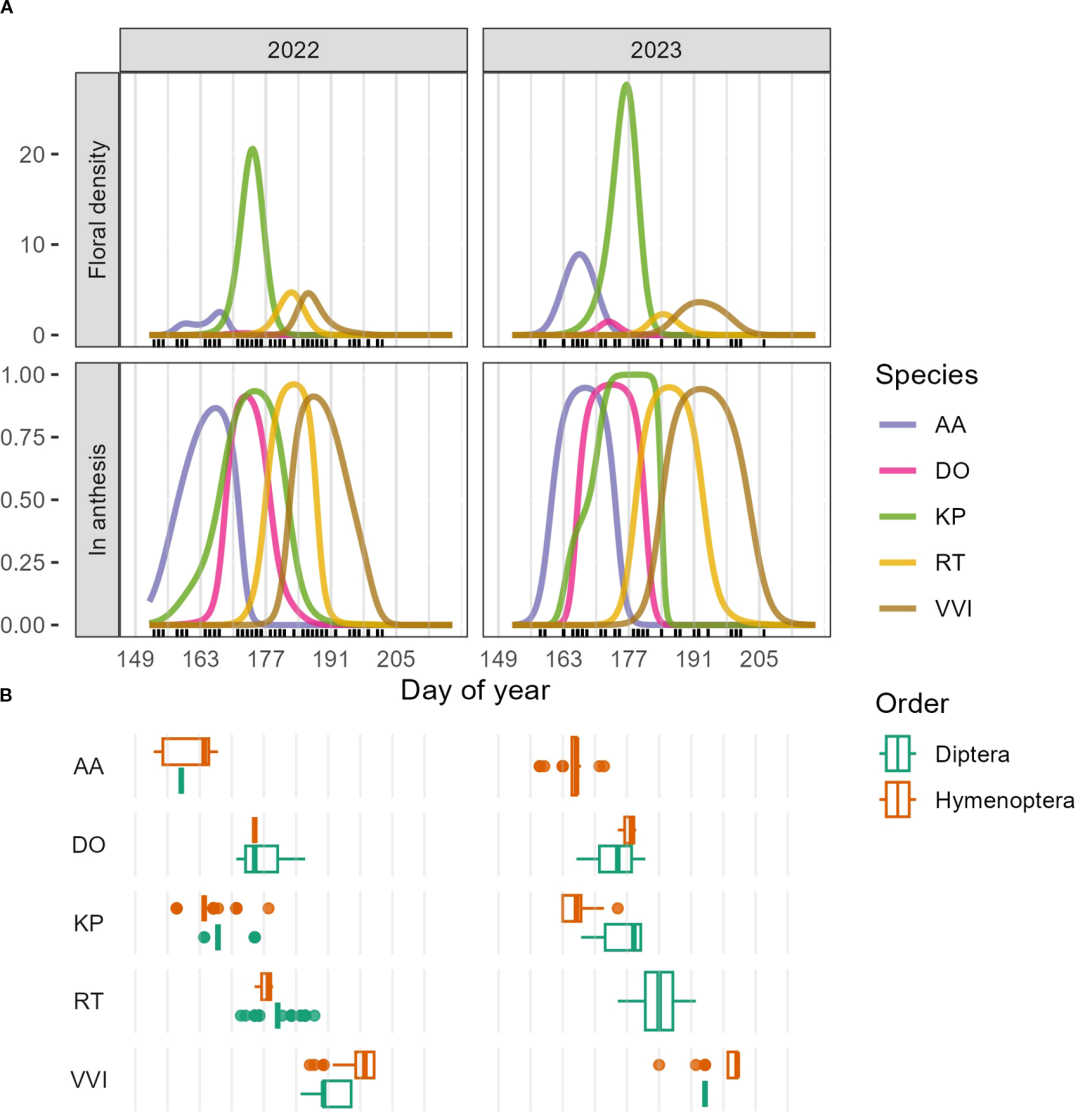
**(A)** GAMM predictions for floral density (first row) and proportion of plots in anthesis (second row) for *Arctous alpina* (AA), *Dryas octopetala* (DO), *Kalmia procumbens* (KP), *Rhododendron tomentosum* (RT), and *Vaccinium vitis-idaea* (VVI). Visitor observations and/or collections were done on days marked with a black tick. Grey vertical lines mark the beginning of each study week. **(B)** Boxplots showing the temporal distribution of insect visits by Dipterans and Hymenopterans to each of the five plant species, including data from insect observations and collections.

Across both sites and years, floral density peaked between weeks five and seven of the growing season (DOY 177-191, June 26-July 10). However, considering each community type, floral density peaked earlier in Dry than in Moist in both years and sites (**Figure 1**, **Supplementary Table 1**). We also found the number of flowering peaks differed between community types and sites. At Imnavait, two flowering peaks occurred in Dry compared to only one peak in Moist (**Figure 1**). In contrast, only one peak occurred in each community at Toolik. Specific species explain these community- and site-level differences. In Dry, *Arctous alpina*, *Dryas octopetala*, and *Kalmia procumbens* explain the first peak in floral density while *Rhodendron tomentosum* and *Vaccinium vitis-idaea* explain the second peak (**Figures 1** and **2A**). One species, *K. procumbens*, was abundant in the dry at Toolik, but absent at Imnavait. The two predominant species driving the flowering peak in the moist were *R. tomentosum* and *V. vitis-idaea*, and to a lesser degree, *Bistorta officinalis.* Anthesis of *B. officinalis* lasted over 60 days in each site. Although we found significant differences in the temporal pattern of mean floral density between plant community types, we did not find that mean flower density, itself, differed between communities in either year or site (**Supplementary Table 1**).

**Figure 3:**
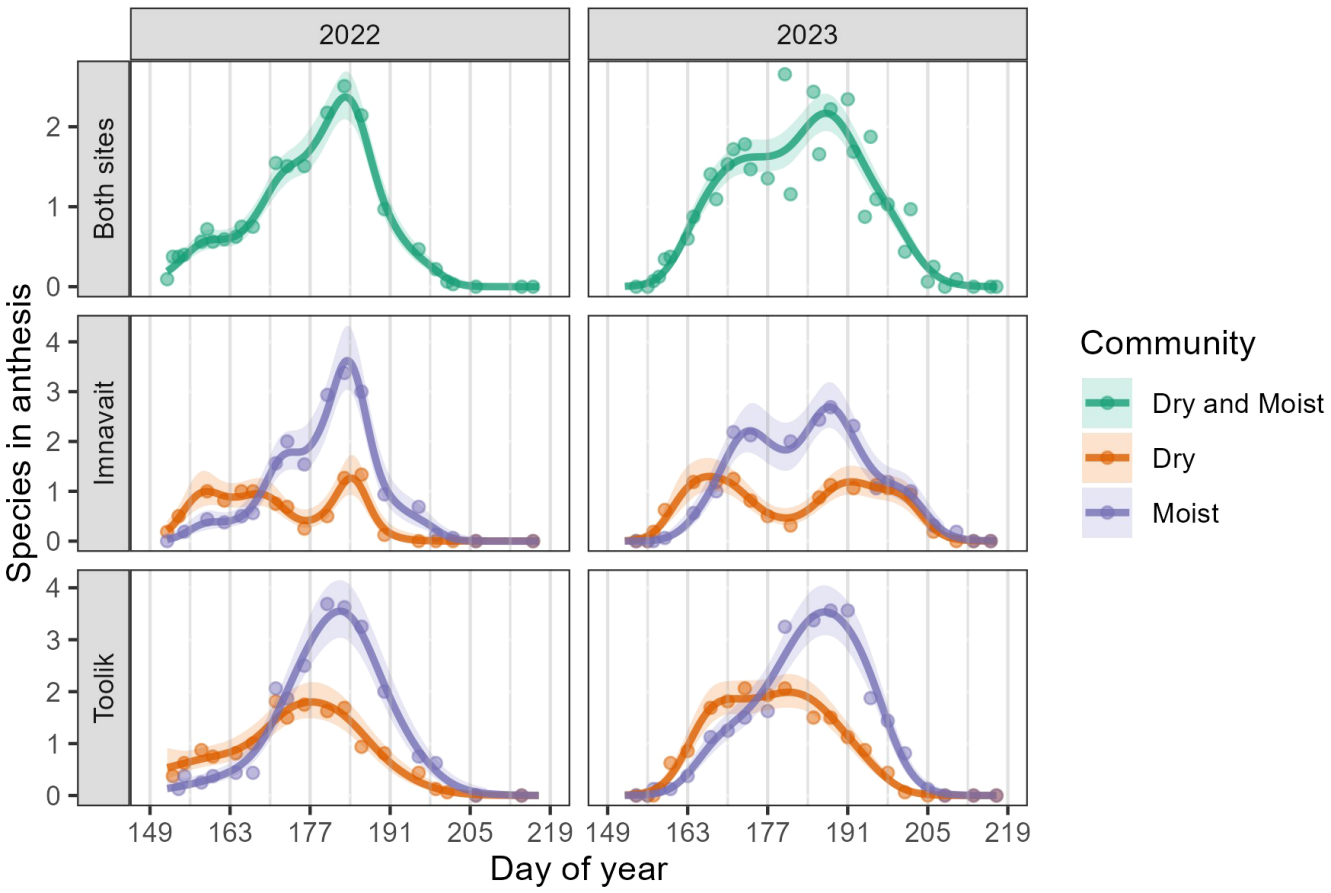
GAMM predictions (smooth trendlines) with 90% confidence bands (shaded) and daily means (points) for the number of species in anthesis per plot. The first row shows Imnavait and Toolik sites combined, while the second and third rows show the individual sites, separated by community type. Grey vertical lines mark the beginning of each study week.

Across both years and sites, the number of species in anthesis peaked in weeks six and seven (DOY 184-190, July 3-July 10). Similar to floral density, the temporal pattern in the number of species in anthesis differed significantly between community types, with Dry peaking before Moist in both years and sites (**Figure 3**, **Supplementary Table 2**). In addition, the number of species in anthesis was significantly higher in Moist (2022: Mean = 1.38, SD = 1.51; 2023: M = 1.24, SD = 1.49) compared to Dry (2022: M = 0.86, SD = 0.89; 2023: M = 0.83, SD = 1.02) in both years at Toolik (2022: z = 2.07, p = 0.04; 2023: z = 6.06, p < 0.001) (**Supplementary Table 2**). We found no significant difference in the mean number of species in anthesis between community types at Imnavait (**Supplementary Table 2**).

### 3.2 Visitation patterns

**Figure 4:**
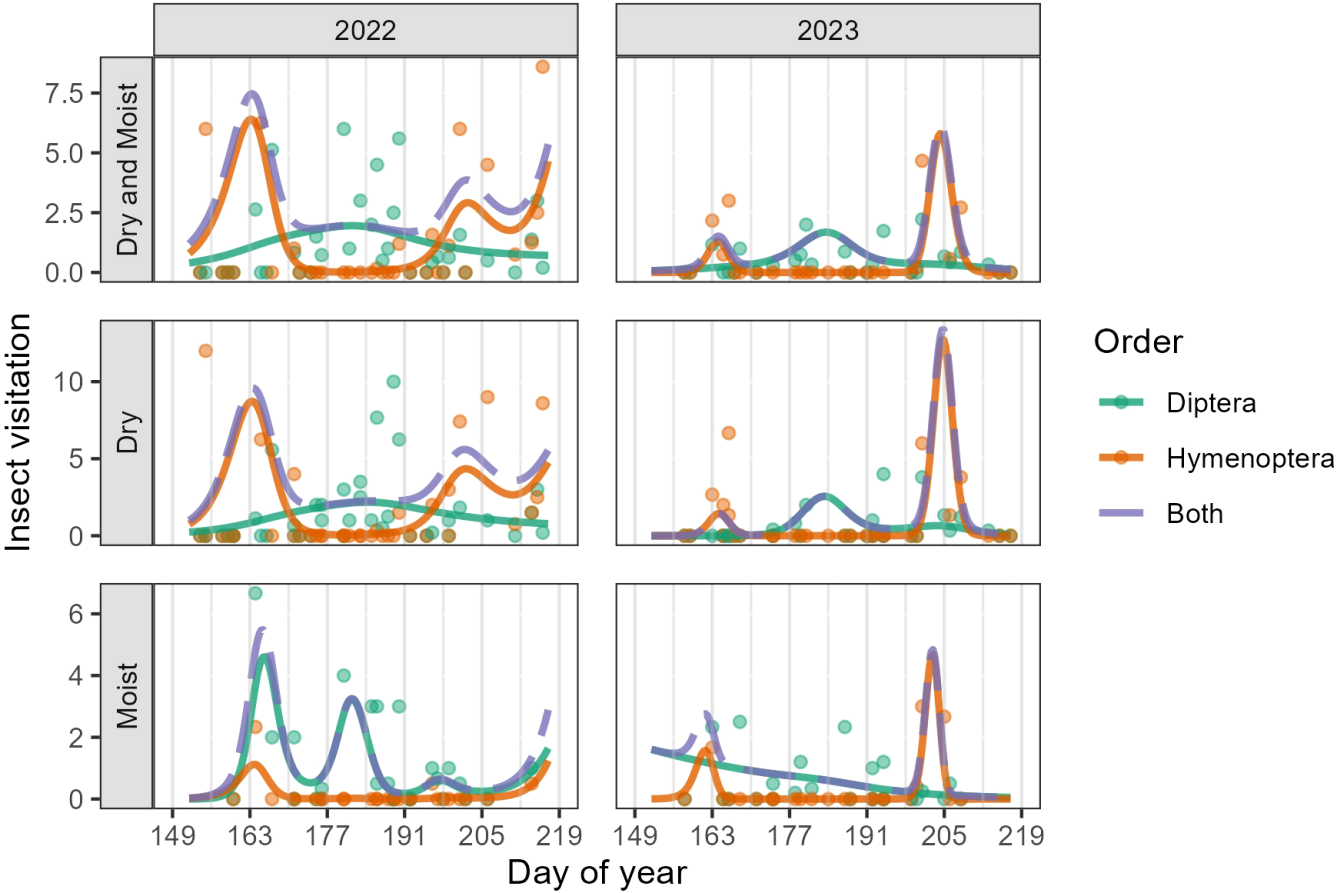
GAMM predictions (smooth trendlines) and daily means (points) for the number of observed insect visits per 10-minute visitor observation. For better visualization, the y-axis was limited not to exceed 13 (a few points were above this value and are not displayed in the plot). Because Dipterans and Hymenopterans made up 98.8% of the insect visits, other orders were excluded from this plot. The first row shows Dry and Moist communities combined, while the second and third rows show the individual communities, separated by visitor order. The dashed trendline (Both) is the sum of predicted visits by Diptera and Hymenoptera. Grey vertical lines mark the beginning of each study week.

Dipterans and Hymenopterans comprised 98.8% of all floral visits and occurred in both communities. Lepidopterans, Coleopterans, Hemipterans, and Trichopterans comprised the remaining 1.2% of visits. In Dry, the temporal pattern of visitation differed significantly between Dipterans and Hymenopterans in both years (2022: F_5.90_ = 5.67, p < 0.001; 2023: F_5.50_ = 2.81, p = 0.01) (**Supplementary Table 3**). In Moist, the temporal pattern of visitation differed between Dipterans and Hymenopterans only in 2022 (F_2.74_ = 3.08, p = 0.03) (see **Supplementary Table 3** for all F statistics and p-values including non-significant values). In both years and community types, Hymenopteran floral visits peaked in the early flowering season between weeks two and four (DOY 156-169, June 5-June 18) and again, between weeks eight and nine (DOY 198-211, July 17-July 30) (**Figure 4**). We recorded very few Hymenopteran visits between weeks four and seven (DOY 170-197, June 19-July 16) and few to no bumblebees (*Bombus* spp.) in the study area during this period. In contrast, visitation by Dipterans showed a more stable pattern, gradually building to a peak beginning in week three and lasting through week seven (DOY 163-197, June 12-July 16). While Hymenopteran and Dipteran activity overlapped to an extent, each order dominated during a different point of the flowering season; Hymenopterans dominated during the early- and late-flowering season, and Dipterans during the mid-flowering season (**Figure 4**). This pattern held true across community types and sites. That is, Hymenopteran activity showed a bimodal peak (early and late season) while Dipteran activity showed a unimodal peak (**Figure 4**).

To connect floral resources and visitation rates, we focused on five plant species for which we had the most floral density and visitation data: *Arctous alpina* (AA), *Dryas octopetala* (DO), *Kalmia procumbens* (KP), *Rhododendron tomentosum* (RT), and *Vaccinium vitis-idaea* (VVI). Flowering occurred sequentially, beginning with the earliest species, *A. alpina*, followed by *D. octopetala* and *K. procumbens*, then *R. tomentosum*, and finally, *V. vitis-idaea* (**Figure 2A**). Flowering in *Arctous alpina* peaked (i.e. flowered in the greatest proportion of plots) between weeks three and four (DOY 163-176) in both study years (**Supplementary Figure 1**). With respect to visitation rates, *A. alpina* was visited almost exclusively by Hymenopterans, specifically bumblebees (97% of visits in 2022 and 100% of visits in 2023) (**Figure 2B**). *Dryas octopetala* peaked between weeks four and five (DOY 170- 183, June 19-July 2) (**Supplementary Figure 2**) and was visited primarily by Dipterans (2022: 97% Dipterans vs 3% Hymenopterans; 2023: 95% Dipterans vs 5% Hymenopterans) (**Figure 2B**). *Kalmia procumbens* peaked between weeks four and five (DOY 170-183, June 19-July 2; **Supplementary Figure 3**) and received visits from both Hymenopterans and Dipterans, although Hymenopterans dominated in both study years (2022: 85% Hymenopterans vs. 15% Dipterans; 2023: 67% Hymenopterans vs. 33% Dipterans) (**Figure 2B**). Interestingly, in 2023 K*. procumbens* received more visits from bees early during flowering, while flies were more common at the end of flowering (**Supplementary Figure 3**). *Rhododendron tomentosum* peaked between weeks five and seven (DOY 177-197, June 26-July 16) (**Supplementary Figure 4**) and was visited exclusively by Dipterans in both years (**Figure 2B**). *Vaccinium vitis-idaea* flowered the latest of the five focal species between weeks six and eight (DOY 184-204, July 3-July 23) (**Supplementary Figure 5**) and was visited primarily by Hymenopterans, although we found substantial interannual variation (2022: 50% Hymenopterans vs. 50% Dipterans; 2023: 96% Hymenopterans vs. 4% Dipterans) (**Figure 2B**).

### 3.3 Plant-insect visitor network

In 2022, we collected 255 insect specimens belonging to 5 orders and 28 families. In 2023, we collected 283 specimens and recorded an additional 127 confirmed visitation events by individual identifiable insects, totaling 410 observed interactions (**Supplementary Table 4**). In both years, the majority of insects belonged to Diptera (69%) and Hymenoptera (24%). Within these orders, the most abundant insect families were Syrphidae, Muscidae, and Apidae. Remaining collections/observations belonged to Order Lepidoptera, Coleoptera, Hemiptera, and Trichoptera (7%). While we constructed our networks at the insect family level, we were able to identify some insect taxa to higher taxonomic levels. For example, we collected eight species of *Bombus* within the Apidae family. *Bombus jonellus* and *B. sylvicola* were the most common species in both years. We also collected individuals of *B. cryptarum, B. johanseni, B. kirbiellus, B. natvigi, B. neoboreus, and B. polaris.* Although Coleopterans comprised a minority of collected specimens (<2%) and recorded visits, lady beetles (Coccinellidae: *Hippodamia arctica*), flower beetles (Cantharidae), and weevils (Curculionidae) were also found in both community types (**Supplementary Table 4**).

#### 3.3.1 2022 cumulative static network

The 2022 cumulative, static network consisted of 41 plant species, 28 insect families, and 128 unique plant-insect links (**Supplementary Figure 6**). All computed network metrices differed significantly from the null models including connectance (C = 0.11, z = 7.94, p< 0.001), nestedness (N = 4.71, z = -2.59, p< 0.001; NODF = 44.61, z = 8.97, p< 0.001), and links per species ( $\bar{L}$ = 1.86, z = 7.60, p< 0.001) (**Table 1**). *Bistorta officinalis, Rhododendron tomentosum*, and *Salix pulchra* were visited by the greatest number of insect families. Syrphidae, Muscidae, and Apidae interacted with the greatest number of plant species.

**Table 1:** Summary of cumulative and sub-set static network indices for each year.

Year	Site	Comm.	Plant	Insect	Links	C	N	NODF	H_2_’	Q	$\bar{L}$
2022	T, I	Cumu.	41	28	128	0.11*	4.71*	44.61*	NA	NA	1.86*
2023	T	Cumu.	26	31	123	0.15*	7.29	45.67	0.34*	0.30*	2.16*
2023	T	Dry	19	22	85	0.20*	10.98	53.50	0.32*	0.31*	2.07*
2023	T	Moist	17	18	51	0.17*	9.26	39.21	0.45*	0.40*	1.46

T, Toolik; I, Imnavait; Comm., community type; Cumu., cumulative; Plant, number of plant species; Insect, number of insect families; Links, total number of links; C, Connectance (0-1); N, Nestedness (0-100); NODF, Nestedness based on Overlap and Decreasing Fill (0-100); H2, Network-level specialization (0-1); Q, Modularity (0-1); L, mean number of links per plant species insect family. H_2_’ and Q values only apply to the weighted (2023) network.*indicates significant difference from null models at p< 0.05.

#### 3.3.2 2023 cumulative static network

**Figure 5:**
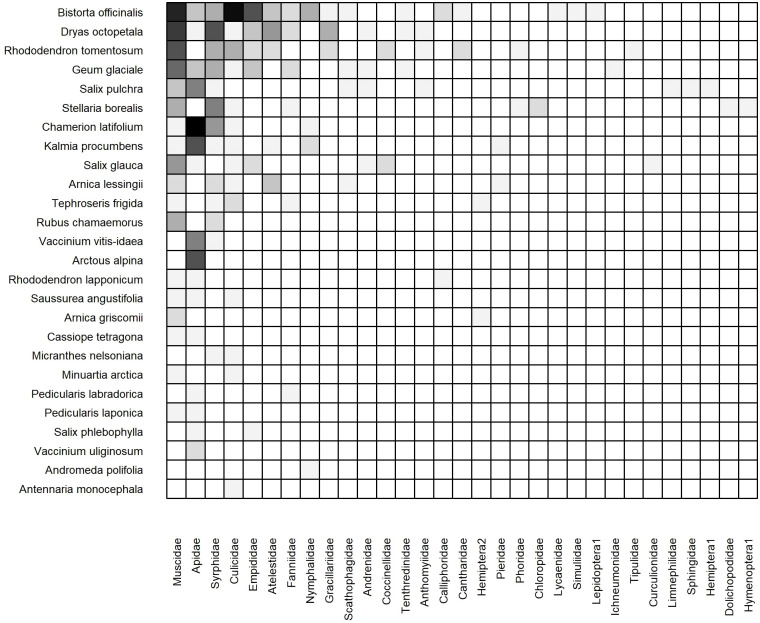
Weighted, cumulative (Dry and Moist combined) static plant-insect visitation matrix based on the log number of visits observed during the 2023 growing season. Plant species are represented on the y-axis and insect families are on the x-axis. A filled box indicates an observed link between a plant and insect. Shading indicates the frequency of the interaction measured by the log number of visits [log(visits+1)]. Each matrix is organized in a nested fashion such that the most generalized species/families occur at the top left and interact with the most partners.

The 2023 weighted, cumulative, static network consisted of 26 plant species, 31 insect families, and 123 unique links (**Figure 5**). Connectance (C = 0.15, z = -5.42, p< 0.001), network-level specialization (H_2_’ = 0.34, z = 10.64, p< 0.001), modularity (Q = 0.30, z = 12.91, p< 0.001) and links per species ( $\bar{L}=$ 2.16, z = -5.1, p< 0.001) all differed significantly from the null models. In contrast, neither metric of nestedness differed significantly from the null models (**Supplementary Table 5**). The core of plant species was similar, but not identical, to that of 2022. Three plant species were visited by the greatest number of insect families: *Bistorta officinalis, Dryas octopetala*, and *Rhododendron tomentosum.* The core of insect families was identical to that of 2022: Muscidae, Apidae, and Syrphidae interacted with the most plant species (**Figure 5**). We found evidence for modularity as several plant species were visited primarily or exclusively by bumblebees (Apidae) including *Arctous alpina, Chamerion latifolium, Kalmia procumbens*, and *Vaccinium vitis-idaea.* In contrast, the core plant species (*B. officinalis, D. octopetala*, and *R. tomentosum*) were primarily visited by Muscidae and Syrphidae, and to a lesser degree other Dipteran families including Culicidae, Empididae, and Fanniidae.

#### 3.3.3 2023 dry and moist static networks

**Figure 6:**
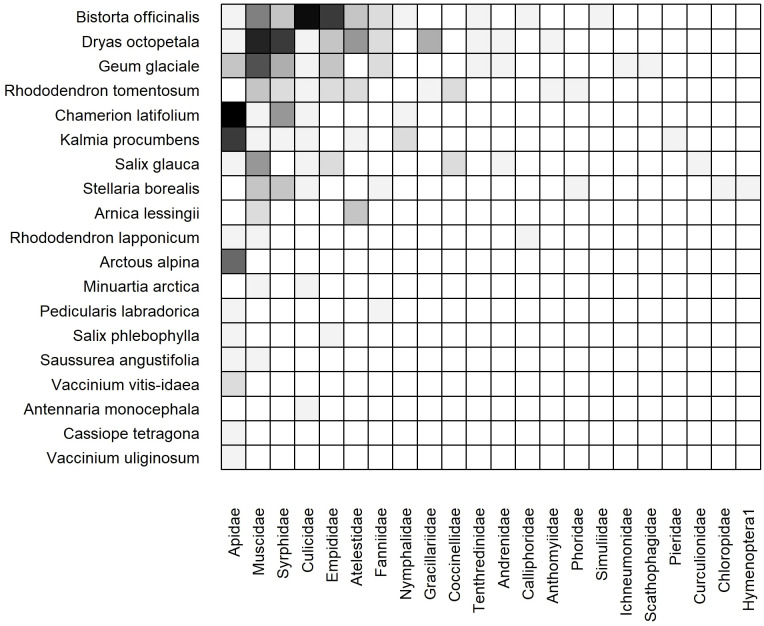
Weighted, subset (dry heath tundra) static plant-insect visitation matrix based on the log number of visits observed during the 2023 growing season in. Plant species are represented on the y-axis and insect families are on the x-axis. A filled box indicates an observed link between a plant and insect. Shading indicates the frequency of the interaction measured by the log number of visits [log(visits+1)]. Each matrix is organized in a nested fashion such that the most generalized species/families occur at the top left and interact with the most partners.

**Figure 7:**
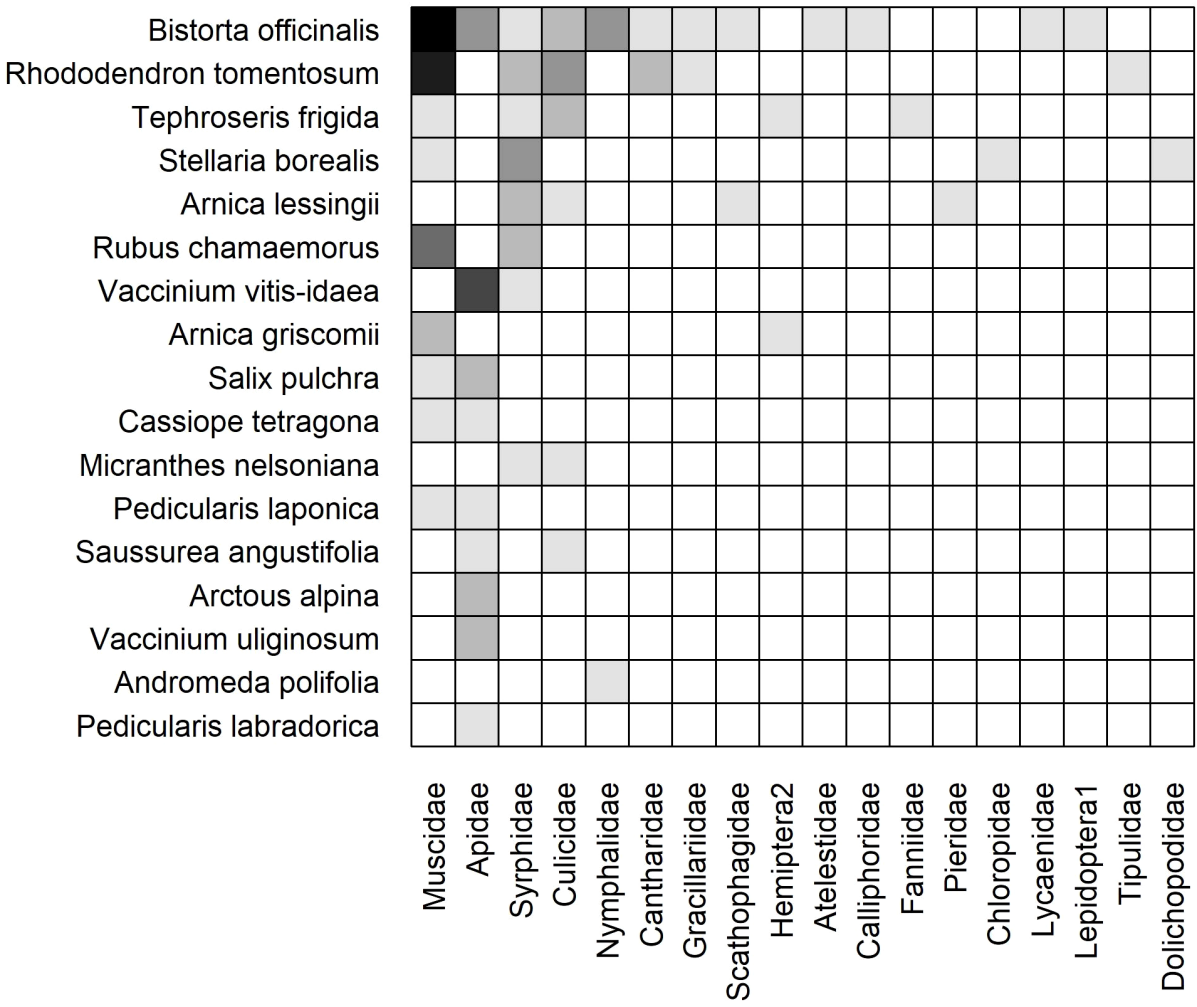
Weighted, subset (moist acidic tundra) static plant-insect visitation matrix based on the log number of visits observed during the 2023 growing season. Plant species are represented on the y-axis and insect families are on the x-axis. A filled box indicates an observed link between a plant and insect. Shading indicates the frequency of the interaction measured by the log number of visits [log(visits+1)]. Each matrix is organized in a nested fashion such that the most generalized species/families occur at the top left and interact with the most partners.

The weighted, static networks for dry (**Figure 6**) and moist (**Figure 7**) communities were structurally similar to the cumulative network. For example, *Bistorta officinalis* and *Rhododendron tomentosum* formed the core of plant species in both networks, although *R. tomentosum* was more generalized in Moist than Dry (i.e. higher frequency of links with a greater number of insect families) (**Figures 6**, **7**). Other core species consisted of *Dryas octopetala* and *Geum glaciale* in Dry and *Tephroseris frigida* and *Stellaria borealis* in Moist.

The dry network included more plant species (19 Dry vs. 17 Moist), insect families (22 vs. 18), and links (85 vs. 51) than the moist network (**Table 1**). Although ten plant species (38%) were active in both communities, nine plant species (35%) were restricted to Dry and seven species (27%) were restricted to Moist (**Figures 6**, **7**).

The core of insect families was also similar in both communities: Apidae, Muscidae, Syrphidae, and Culicidae. However, Apidae was more generalized in Dry owing to frequent interactions with plant species that occur exclusively in the dry community such as *Chamerion latifolium*, *Kalmia procumbens*, and *Arctous alpina* (**Figure 6**). Twelve insect families were collected in both communities (39%) while ten (32%) and six (19%) were only found in Dry and Moist, respectively. For example, Empididae was only found in Dry and Dolichopodidae was only found in Moist (**Figures 6**, **7**).

Connectance, specialization, and modularity differed significantly from those of null models in both communities (Dry C = 0.20, z = -3.95, p< 0.001, Moist C = 0.17, z = -1.87, p< 0.05; Dry H_2_’ = 0.32, z = 11.84, p< 0.001, Moist H_2_’ = 0.45, z = 2.05, p< 0.05; Dry Q = 0.31, z = 12.21, p< 0.001, Moist Q = 0.40, z = 2.93, p< 0.01). In contrast, neither metric of nestedness differed significantly from those of the null models in either community. Links per species differed significantly from those of the null models only in Dry ( $\bar{L}=$ 2.07, z = -3.72, p< 0.001).

#### 3.3.4 Temporal patterns and 2023 dynamic networks

**Figure 8:**
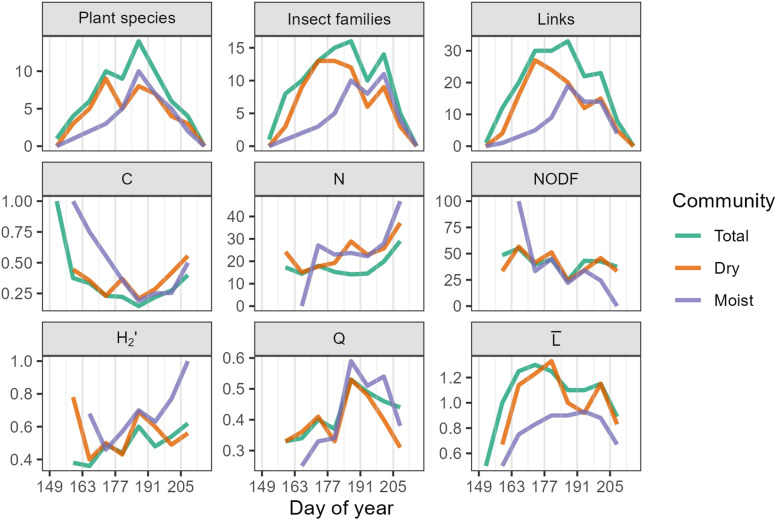
Network indices for the 2023 dynamic network. Grey vertical lines mark the beginning of each study week. Total refers to the cumulative dynamic network, Dry and Moist refer to the subset dynamic networks. C = Connectance (range 0-1), N = nestedness (0-100), NODF = Nestedness based on Overlap and Decreasing Fill (0-100), H_2_’ = network-level specialization (0-1), Q = quantitative modularity (-1-1), and $\bar{L}$ = mean number of links per plant species-insect family.

Over the growing season, we observed a buildup and decline in network size reflected through the number of plant species, insect families, and links. The size of the cumulative, dynamic network gradually increased until a peak during week six (DOY 184-190, July 3-July 9), then decreased over the next four weeks (**Figure 8**). In this way, the duration of network buildup was longer than the duration of network decline. We also found that the number of plant species and links in Dry peaked during week four (DOY 170-176, June 19-June 25) compared to week six (DOY 184-190, July 3-July 9) in Moist. The temporal pattern in the number of insect families was even more distinct between communities; Dry peaked in week four (DOY 170-176, June 19-June 25) and Moist peaked in week eight (DOY 198-204, July 17-July 23) (**Figure 8**).

In the cumulative network, connectance peaked at the beginning and end of the season, reaching a minimum during week six (DOY 184-190, July 3-July 9) (**Figure 8**, **Supplementary Table 5**). Nestedness was highest between weeks two and four (DOY 156-176, June 5-June 25), and again during weeks eight and nine (DOY 198-212, July 17-July 31). Network-level specialization (H_2_’) increased slightly over the growing season, while modularity (Q) peaked during week six (DOY 184-190, July 3-July 9). The mean number of links ( $\bar{L}$ ) showed a gradual increase and decline, peaking in week four (DOY 170-176, June 19-June 25). Network metrices for the dry and moist communities often mirrored trends in the cumulative community. However, the moist community had higher H_2_’ and Q, and lower $\bar{L}$ than the dry. These patterns in specialization and modularity were most pronounced at the end of the growing season (**Figure 8**, **Supplementary Table 5**).

### 3.4 Sampling estimates

In both the cumulative and subset networks, sampling completeness and coverage were higher for plants and visitors than links (**Supplementary Figures 7 and 8**). Specifically in the 2023 cumulative static network, sampling coverage was highest for plants (99%) and visitors (97%), and lowest for links (84%). In other words, we sampled 99% of the plants and 97% of the floral visitors present, but only 84% of the interactions. Similarly, median sampling coverage by week in the cumulative dynamic network was higher for plants (98%) and visitors (90%) than links (76%).

Sampling coverage in the subset static networks followed the same pattern: Dry plants (99%) and Dry visitors (97%) vs. Dry links (82%), Moist plants (99%) and Moist visitors (92%) vs. Moist links (72%). Median sampling coverage by week showed consistently higher coverage in Dry compared to Moist for plants (Dry = 96% vs. Moist = 86%), visitors (90% vs. 84%), and links (77% vs. 60%) (**Supplementary Figure 8**). From a temporal perspective, sampling coverage was more constant over the growing season for plants and visitors than links. That said, sampling coverage of plants, visitors, and links was lowest when network size peaked (weeks 6-7) (**Supplementary Figure 8**). These results suggest that our ability to capture the entire network was limited when the greatest number of plant species were in bloom.

## 4 Discussion

We tested three research questions examining floral resources, visitation rates, and network structure in two tundra plant community types. We found temporal differences in floral availability and visitation rates between community types. Network buildup also differed between communities, with the dry peaking before the moist.

### 4.1 Spatio-temporal patterns of floral resources

Floral density and the number of species in anthesis peaked earlier in Dry compared to Moist. We attribute differential timing in floral abundance to both abiotic conditions and community composition. Snow depth and duration are the most important factors differentiating tundra plant communities as they determine other important abiotic variables including soil temperature and moisture, thaw depth, and resource availability (Molau, 1993). The dry community is characterized by less snow accumulation and earlier snowmelt than the moist (Walker et al., 1994).

Timing of snowmelt and depth of snow are related to flowering phenology and abundance (Wipf and Rixen, 2010; Oberbauer et al., 2013; Semenchuk et al., 2016). For example, in the High Arctic, Bjorkman et al. (2015) found a strong relationship between timing of snowmelt and flowering phenology, particularly in early-flowering species. Species that occur in dryer habitats are exposed to lower snow cover and earlier melt-out than those growing in moister habitats. Physiological characteristics of early-flowering species may render them more frost hardy and able to flower in colder temperatures, characteristic of the early season (Semenchuk et al., 2013). In our sites, peak floral resource abundance in the dry community was driven by early-flowering species including *Arctous alpina* and *Dryas octopetala*. Such species can tolerate shallow and inconsistent snow cover during winter (Walker et al., 1994). Thus, early flowering species are adapted to early snowmelt associated with the dry community and drive the first flowering peak we observed.

In addition to snow regime, community composition explains differential timing in floral abundance. For example, within the dry community type, we observed two defined flowering peaks at the Imnavait site compared to one broader peak at the Toolik site. The evergreen shrub, *Kalmia procumbens*, helps explain this difference. At Toolik, *K. procumbens* flowered between early flowering species such as *Arctous alpina* and mid- to late flowering species such as *Vaccinium vitis-idaea*, consequently bridging the two flowering peaks. We did not see this pattern at Imnavait due to the absence of *K. procumbens*. Thus, the presence of a single species, especially one that has high floral density such as *K. procumbens*, can influence the community’s flowering peak.

With respect to the quantity and type of flowers, temporal differences between community types may result in more consistent floral resource availability for mobile pollinators than if flowering occurred synchronously. Differences in timing of snowmelt and heterogeneous snow depth across the landscape can lead to an overall longer flowering duration and increased temporal availability of resources (Gillespie and Cooper, 2022). In our study, important floral resources for insects in the early season (based on floral density) were *Arctous a*lpina, *Dryas octopetala*, *Kalmia procumbens*, and *Salix pulchra* in the dry heath. These species produce valuable nectar and pollen resources for floral visitors (Williams and Batzli, 1982; Tiusanen et al., 2016; Khorsand et al., 2024). As flowering waned in these species, other species such as *Rhododendron tomentosum*, *Bistorta officinalis*, and *Vaccinium vitis-idaea* became increasingly important. All three of these species are considered mid- to late-flowering species at our sites and occur in both community types, corresponding with the second peak in the dry and the broad, singular peak in the moist. Thus, heterogeneity in the timing and taxa of floral resources lengthens the overall duration of resources available to insects. This turnover is important because the flowering season is inherently short and the duration of the season appears to be contracting in the High Arctic (Høye et al., 2013), although this pattern has not yet been empirically demonstrated in the Low Arctic. Given the potential for a shorter growing season, floral resource heterogeneity may provide a critical refuge for the pollinator community.

### 4.2 Insect visitor community and visitation rates

Dipterans and Hymenopterans accounted for the majority of observed visits and collected specimens. However, visitation by each insect order varied temporally over the growing season. Hymenopterans, predominantly bumblebees (*Bombus* spp.), showed bimodal visitation activity at the beginning and end of the season while Dipteran visitation was more stable and unimodal. Closely linked plant-pollinator phenology may explain the temporal differences in visitation activity (Bartomeus et al., 2011 and 2013). Bumblebees depend on floral resources for their entire life cycle in contrast to flies, whose larval diet does not depend on floral resources (Forrest, 2015; Raguso, 2020). In early spring, solitary queens emerge from overwintering diapause to forage and garner resources to produce broods of workers, then finally males and new queens (Williams et al., 2024). Near Atkasook, Alaska, queen bumblebees were observed in late May/early June, followed by workers in early to mid-July, and finally males and new queens in late July/early August (Williams and Batzli, 1982). In our study, we observed a similar temporal pattern of bumblebee activity aligning with caste emergence. Our collections and visitor observations indicate that emerging queens of multiple *Bombus* species feed on *A. alpina* and *K. procumbens* early in the season, presumably to support the production of eggs. We propose that the lull in bumblebee visitation during the mid-season flowering peak may correspond with the incubation of the first worker brood, during which time queen bumblebees are still responsible for maternal care of larvae in the nest (Gustilo et al., 2023). Then, during the mid- and late-season, *V. vitis-idaea* and *C. latifolium* provide critical resources for emerging workers, and later, males and new queens. In contrast, Dipteran larval development does not rely on floral resources, which may explain its stable activity through the growing season (Forrest, 2015; Raguso, 2020).

At the family level, muscid flies were more abundant (comprising the majority of insect collection) and collectively responsible for more links than any other insect family. Other Dipteran families including Syrphidae, Empididae, Fanniidae, and Culicidae were also abundant and generalized floral visitors. These findings corroborate other High Arctic and alpine studies that point to Dipterans, especially muscids and syrphids, as dominant floral visitors (Lundgren and Olesen, 2005; Robinson et al., 2018; Tiusanen et al., 2016). In addition to their ability to transport pollen (Pont, 1993; Skevington and Dang, 2002), Muscids are active for a long period of the growing season (Cirtwill et al., 2023), increasing the probability that they serve as effective pollinators in the Arctic. That said, our results demonstrate that bumblebees (Apidae) are also an important part of the plant-pollinator network on Alaska’s North Slope. In contrast to High Arctic studies in which bumblebees are rare (Elberling and Olesen, 1999; Olesen et al., 2008; Robinson et al., 2018) or absent (Burns et al., 2022; Gillespie and Cooper, 2022), our network was relatively species rich containing eight species of *Bombus*. Bumblebees carry significantly higher pollen loads than any other insect family with the exception of Muscidae (Khorsand et al., 2024; Khorsand unpublished data), suggesting they may be effective pollinators at our sites. Future research quantifying pollen loads on floral visitors and temporal patterns in pollen transport is necessary to determine the role of Muscidae and Apidae in our network.

### 4.3 Network complexity and structure

Temporal dynamics in flowering strongly influence network structure, particularly for networks that are sampled at a broader scale (weeks to months) (Schwarz et al., 2020), such as ours. We observed that network size, floral abundance and richness, as well as the number of insect families active in the cumulative network all peaked simultaneously. Network size parameters peaked earlier in the dry community compared to the moist community, also matching the temporal patterns in floral resource availability we recorded in the two plant community types. Previous studies in temperate and alpine systems have shown alignment between network structure and weekly floral changes (Burkle and Alarcón, 2011; Simanonok and Burkle, 2014), suggesting that changes in the network mirror changes in the plant community. In the High Arctic, Robinson et al. (2018) found that flower diversity in Nunavut was a stronger predictor of network complexity than the insect community. Gillespie and Cooper (2022) described build-up in Svalbard network complexity coinciding with peak flower production and insect visitation rates, followed by a period of network “stasis” as plant senescence occurred. In contrast, Pradal et al. (2009) found that the Greenlandic network collapsed at the end of the season instead of declining gradually, perhaps in response to the abiotic environment. That said, the authors also found a very high correlation between the disappearance of pollinators and disappearance of plants from the network. These studies, in conjunction with our current study, point to the diversity and abundance of floral resources as major drivers of network timing and complexity. Although abiotic factors such as air temperature influence plant phenology, biotic factors such as biodiversity may be more important in shaping network structure over time (Robinson et al., 2018).

The 2022 and 2023 cumulative static networks exhibited compositional and structural similarities despite sampling at different spatial scales. We restrict our interpretation of network metrices to 2023, as the 2022 network was constructed from opportunistic collections. Nevertheless, the shared taxonomic similarity between years highlights the consistency in plant-visitor interactions and community composition.

We found some key structural differences in our static networks compared to other Arctic plant species-insect family networks. First, our 2023 cumulative static network had higher plant and insect richness than observed networks in Abisko, Sweden (Elberling and Olesen, 1999), Uummannaq, Greenland (Lundgren and Olesen, 2005), Alexandra Fjord, Canada (Robinson et al., 2018), and Adventdalen, Svalbard (Gillespie and Cooper, 2022), all of which are at higher latitudes than our sites (see Table 2 in Gillespie and Cooper, 2022, but also see Olesen et al., 2008). Species richness of both plants and insects is expected to decrease with latitude (Willig, 2000; Orr et al., 2020). Second, our network exhibited lower connectance than all aforementioned High Arctic networks. Network size and connectance are inversely related (Olesen and Jordano, 2002), suggesting that in more speciose networks, fewer total possible links will be realized. We sampled in two community types, capturing higher plant species richness and consequently, a larger network. In addition, high temporal turnover of plant species can result in low network connectance. A network that is active for longer than one month will inevitably include plant species with non-overlapping phenologies (Basilio et al., 2006), leading to forbidden links, or links that cannot form because species are temporally separated (Olesen et al., 2011). Thus, a large network sampled over months and characterized by high species turnover will likely have lower connectance (Burkle et al., 2013), as we found at our sites. That said, we acknowledge that network size could be even larger and connectance lower if we accounted for insect species instead of family. Future plant-pollinator network studies are warranted in the Arctic, specifically at higher taxonomic resolution of insects.

While true specialization is rare in Arctic networks (Olesen and Jordano, 2002), our H_2_’ values were significantly higher than null model expectations. Other Arctic studies do not necessarily compute this network metric (Gillespie and Cooper, 2022), complicating a direct comparison. However, additional specialization metrices such as links per species were lower in our network (i.e. more specialized) than these studies. These findings suggest that while our network appears more specialized than other documented High Arctic networks, it is more generalized at the taxonomic level we examined compared to temperate and tropical networks (Olesen and Jordano, 2002). High generalization is expected at high latitudes (Olesen and Jordano, 2002). While our nestedness metrices did not significantly differ from null models, we observed moderate values and typical patterns of nestedness including a generalized ‘core’ and two ‘tails’ of more specialized plant species and insect families (Olesen et al., 2008). Other Arctic network studies have also reported high generalization and nestedness, corroborating our findings (Elberling and Olesen, 1999; Lundgren and Olesen, 2005; Olesen et al., 2008; Robinson et al., 2018; Gillespie and Cooper, 2022).

Within the generalized network, specific modules characterized by more specialized interactions emerged. Although our Q values for both the cumulative and subset static networks were moderate, modularity in these networks significantly exceeded the null models. High plant species turnover contributes to the formation of modules as temporal separation among plant species can lead to more specialized interactions (Schwarz et al., 2020). In addition, the presence of bumblebees (*Bombus* spp.) helps explain module formation in our network. Bumblebees dominated or were exclusively responsible for recorded visits to these four plant species: *Arctous alpina*, *Kalmia procumbens*, *Vaccinium vitis-idaea*, and *Chamerion latifolium*. Sequential flowering of these species provides consistent foraging resources for bumblebees over the growing season, thereby allowing the module to persist. In contrast to Apidae which showed strong preference for specific species and formed a clear module, we observed that the most abundant fly families had more generalized preferences and did not form clear modules. Thus, the turnover of floral resources coupled with the presence of specific pollinators and their unique life history may act synergistically to shape network structure.

Sampling approach and effort may also explain the observed patterns in connectance, specialization, and modularity. Any network study must acknowledge the difference between the ‘true’ network and the ‘observed structure’ (Vázquez et al., 2009), as the latter is inherently influenced by sampling effort and bias (Vázquez and Aizen, 2006; Jordano, 2016). Both species abundance and flowering duration can lead to oversampling some links more than others. In a generalist network, the most abundant species will have the highest frequency of interactions with the highest number of species. These abundant species will, therefore, appear more generalized than rare species. Other Arctic studies have reported that the most abundant plant and insect species with the longest phenophases shared the most interactions with other taxa (Olesen et al., 2008; Gillespie and Cooper, 2022). Conversely, rare species tend to comprise the specialized tails of the network (Olesen et al., 2008). We utilized a focal plant species observation method in 2023 to reduce the sampling bias towards abundant species. However, we observed species with longer phenophases more than species with short phenophases, which may contribute to the specialized tails in our network and corresponding low connectance. That said, our sampling coverage estimations demonstrate that we sampled the majority of links and more than 90% of plants and floral visitors. Unobserved links may be less critical in a seasonal network than in a short-term network (Schwarz et al., 2020).

One plant species, in particular, stands out as a key resource for floral visitors both from a spatial and temporal perspective. In the dry and moist networks, *Bistorta officinalis* formed the core of each network, attracted the most insect families, and received the most visits. Goldstein and Zych (2016) consider *B. officinalis* to be a “hub species” because it is a core resource for the insect visitor community. Furthermore, *B. officinalis* is the longest flowering species at our sites. Plants with longer phenophases tend to accumulate more links over time, leading to lower network specialization (Schwarz et al., 2020). Thus, as one of the core species, *B. officinalis* contributes substantially to the number of links and connectance in both dry and moist tundra, and may function as a network “connector” (Gonzalez et al., 2010). In addition, *B. officinalis* has been shown to be pollen-limited (Khorsand et al., 2024). Given this species’ dependence on floral visitors for fruit set, a long flowering phenophase may increase the number of interactions with floral visitors and facilitate reproductive success of this species.

### 4.4 Network resiliency in a warming Arctic

Spatio-temporally dynamic systems may promote network resiliency (Caradonna et al., 2017). Heterogeneity in floral resources, flexibility in resource use by insects, and overall diversity in network structure permit ‘rewiring’ of the network (Cirtwill et al., 2023). Previous studies have underscored the critical role rewiring plays in maintaining community structure and stability (Olesen et al., 2008; Kaiser-Bunbury et al., 2010; Staniczenko et al., 2010; Caradonna et al., 2017). As the intensity and frequency of extreme weather events become more common in the Arctic (Landrum and Holland, 2020), variation in habitat-specific abiotic conditions may increase, potentially altering plant community composition and foraging resources for insects. Low species diversity presents another challenge to Arctic networks in the face of climate change (Vasiliev and Greenwood, 2021). Habitat diversity and asynchronous flowering may buffer species from abiotic stressors and expand niche availability for plant-insect interactions to persist (Burkle et al., 2013; Carvell et al., 2017). In our study, network timing, size, and structure differed between the dry and moist communities, with each community offering favorable foraging habitat at different points of the growing season. Thus, spatio-temporal variation in floral resources and network generalization have the potential to protect the network from ongoing environmental extremes and disturbance. Even temporally co-occurring species can rewire the links of a network by switching interaction partners over time (Poisot et al., 2012). However, the core must remain stable to ensure the integrity and resiliency of the network (Cirtwill et al., 2023). A decline in a few core plant species and/or a few visitor families could affect the entire network. Thus, we argue that the existing diversity of our network, albeit low in a global context, is critical to its own persistence.

## 5 Conclusions

In conclusion, we show temporal differences in floral resource availability between plant community types. While the overall network was generalized with specific cases of modularity, we found temporal differences in the buildup and decline of network structure in each community corresponding with floral resource availability. These findings suggest that habitat variation is critical to the integrity of the plant-pollinator network and may buffer the system against the rapid changes associated with anthropogenic warming. Both bumblebees and muscid flies were key to the network, but had temporally distinct visitation rates. Given the importance of bumblebees as visitors to numerous plant species in the network and their short pulses of activity corresponding with life history, we emphasize the need for further research on bumblebee pollination in the Alaskan Arctic. This is particularly important because bumblebees form a specialized module within the generalized network. Consequently, bumblebee-pollinated plant species may be more susceptible if plant-pollinator mismatch occurs under the warming scenario.

## Data Availability

The raw data supporting the conclusions of this article will be made available by the authors, without undue reservation.
