# Supplementary material for: Spatio-temporal patterns in floral resources and plant-pollinator network structure in the Alaskan Arctic
Source: Front Plant Sci. 2025 Sep 24;16:1552422. doi: 10.3389/fpls.2025.1552422 (PMC12504194; doi:10.3389/fpls.2025.1552422)
Supplement: Supplementary file 1 [file DataSheet1.pdf]

Supplemental Table 1. GAMM summaries for plot floral density. The summaries were obtained with the following R syntax for each combination of year and location: summary (mcgv::gam(FD ~ Community + s(Day) + s(Day, by = Community) + s(PlotU, bs = 're'), family = tw(), method = "REML", data = filter(data, Year == …, Location == …))). The models were set to have treatment contrasts for community type, with Dry as the reference level.

**Year = 2022, Location = Imnavait**				
Family: Tweedie (p = 1.284), Link: log, Deviance explained: 62.6%				
**Parametric term**	**coefficient**	**SE**	**t**	**p-value**
Intercept	-5.530	2.64	-2.09	0.037
Moist	2.335	2.23	1.05	0.296
**Spline**	**edf**	**Ref.df**	**F**	**p-value**
s(Day)	7.27	7.64	8.35	<0.0001
s(Day): Moist	5.29	6.14	8.80	<0.0001
s(Plot)	20.99	30.00	2.79	<0.0001
**Year = 2022, Location = Toolik**				
Family: Tweedie (p = 1.372), Link: log, Deviance explained: 61.8%				
**Parametric term**	**coefficient**	**SE**	**t**	**p-value**
Intercept	-5.54	0.48	-1.12	0.263
Moist	-0.40	0.52	-0.77	0.440
**Spline**	**edf**	**Ref.df**	**F**	**p-value**
S(Day)	6.89	7.72	13.85	<0.0001
S(Day): Moist	3.10	3.85	12.99	<0.0001
S(Plot)	24.58	30.00	3.77	<0.0001
**Year = 2023, Location = Imnavait**				
Family: Tweedie (p = 1.296), Link: log, Deviance explained: 68.2%				
**Parametric term**	**coefficient**	**SE**	**t**	**p-value**
Intercept	-1.58	0.59	-2.67	0.008
Moist	-1.45	0.74	-1.96	0.051
**Spline**	**edf**	**Ref.df**	**F**	**p-value**
S(Day)	7.86	8.40	16.98	<0.0001
S(Day): Moist	4.56	5.45	11.20	<0.0001
S(Plot)	24.13	30.00	3.64	<0.0001
**Year = 2023, Location = Toolik**				
Family: Tweedie (p = 1.401), Link: log, Deviance explained: 64.8%				
**Parametric term**	**coefficient**	**SE**	**t**	**p-value**
Intercept	-2.35	0.76	-3.10	0.002
Moist	0.20	0.36	0.57	0.567
**Spline**	**edf**	**Ref.df**	**F**	**p-value**
S(Day)	7.18	7.82	23.96	<0.0001
S(Day): Moist	1.00	1.00	72.62	<0.0001
S(Plot)	24.36	30.00	3.62	<0.0001

Supplemental Table 2. GAMM summaries for the number of species in anthesis per plot. The summaries were obtained with the following R syntax for each combination of year and location: summary (mcgv::gam(in_bloom ~ Community + s(Day) + s(Day, by = Community) + s(PlotU, bs = 're'), family = poisson(), method = "REML", data = filter(data, Year == …, Location == …))). The models were set to have treatment contrasts for community type, with Dry as the reference level.

**Year = 2022, Location = Imnavait**				
Family: Poisson, Link: log, Deviance explained: 64.2%				
**Parametric term**	**coefficient**	**SE**	**z**	**p-value**
Intercept	-2.97	0.89	-3.32	0.0009
Moist	1.53	0.83	1.85	0.06
**Spline**	**edf**	**Ref.df**	X^2	**p-value**
s(Day)	6.94	7.47	37.80	<0.0001
s(Day): Moist	5.24	6.12	68.49	<0.0001
s(Plot)	3.75	30.00	4.34	0.26
**Year = 2022, Location Toolik**				
Family: Poisson, Link: log, Deviance explained: 63.1%				
**Parametric term**	**coefficient**	**SE**	**z**	**p-value**
Intercept	-0.83	0.16	-5.29	<0.0001
Moist	0.35	0.17	2.07	0.04
**Spline**	**edf**	**Ref.df**	X^2	**p-value**
S(Day)	5.34	6.42	85.26	<0.0001
S(Day): Moist	2.37	2.98	42.52	<0.0001
S(Plot)	10.24	30.00	15.92	0.03
**Year = 2023, Location Imnavait**				
Family: Poisson, Link: log, Deviance explained: 67%				
**Parametric term**	**coefficient**	**SE**	**z**	**p-value**
Intercept	-1.27	0.19	-6.71	<0.0001
Moist	-0.07	0.25	-0.27	0.79
**Spline**	**edf**	**Ref.df**	X^2	**p-value**
S(Day)	7.49	8.26	67.97	<0.0001
S(Day): Moist	4.44	5.40	40.05	<0.0001
S(Plot)	0.01	30.00	0.01	0.59
**Year = 2023, Location Toolik**				
Family: Poisson, Link: log, Deviance explained: 71.7%				
**Parametric term**	**coefficient**	**SE**	**z**	**p-value**
Intercept	-1.83	0.25	-7.31	<0.0001
Moist	0.53	0.09	6.06	<0.0001
**Spline**	**edf**	**Ref.df**	X^2	**p-value**
S(Day)	6.65	7.51	237.39	<0.0001
S(Day): Moist	1.00	1.00	57.34	<0.0001
S(Plot)	0.00	30.00	0.00	0.79

Supplemental Table 3. GAMM summaries for the number of insect visits per 10-minute observation. The summaries were obtained with the following R syntax for each combination of year and community type: summary (mcgv::gam(Landings ~ Order + s(Day) + s(Day, by = Order) + s(PlotU, bs = 're'), family = tw(), method = "REML", data = filter(data, Year == …, Community == …))). The models were set to have treatment contrasts for insect order, with Diptera as the reference level.

**Year = 2022, Community = Dry**				
Family: Tweedie (p = 1.40), Link: log, Deviance explained: 51.6%				
**Parametric term**	**coefficient**	**SE**	**t**	**p-value**
Intercept	0.23	0.22	1.05	0.30
Hymenoptera	-0.05	0.32	-0.17	0.87
**Spline**	**edf**	**Ref.df**	**F**	**p-value**
s(Day)	2.65	3.21	1.50	0.21
s(Day): Hymenoptera	5.90	6.91	5.67	<0.0001
s(Plot)	29.43	108.00	0.38	0.02
**Year = 2022, Community = Moist**				
Family: Tweedie (p = 1.01), Link: log, Deviance explained: 88.5%				
**Parametric term**	**coefficient**	**SE**	**t**	**p-value**
Intercept	-0.31	0.28	-1.13	0.26
Hymenoptera	-3.37	0.71	-4.74	<0.0001
**Spline**	**edf**	**Ref.df**	**F**	**p-value**
S(Day)	6.98	7.57	3.91	0.0005
S(Day): Hymenoptera	2.74	3.30	3.08	0.03
S(Plot)	19.92	30.00	2.35	<0.0001
**Year = 2023, Community = Dry**				
Family: Tweedie (p = 1.43), Link: log, Deviance explained: 75%				
**Parametric term**	**coefficient**	**SE**	**t**	**p-value**
Intercept	-2.08	0.73	-2.85	0.005
Hymenoptera	-3.94	2.86	-1.38	0.17
**Spline**	**edf**	**Ref.df**	**F**	**p-value**
S(Day)	4.45	5.35	2.50	0.04
S(Day): Hymenoptera	5.50	6.36	2.81	0.01
S(Plot)	18.90	62.00	0.69	0.0001
**Year = 2023, Community = Moist**				
Family: Tweedie (p = 1.27), Link: log, Deviance explained: 69.2%				
**Parametric term**	**coefficient**	**SE**	**t**	**p-value**
Intercept	-0.85	0.39	-2.16	0.03
Moist	-8.54	8.91	-0.96	0.34
**Spline**	**edf**	**Ref.df**	**F**	**p-value**
S(Day)	1.60	1.93	2.09	0.11
S(Day): Hymenoptera	4.64	5.31	0.87	0.39
S(Plot)	16.21	39.00	0.91	0.001

Supplemental Table 4. Taxonomic diversity and sample sizes of collected insects by order and family.

Order & Family	2022	2023	Total
**Coleoptera**	**1**	**9**	**10**
Cantharidae	1	3	4
Coccinellidae	0	5	5
Curculionidae	0	1	1
**Diptera**	**182**	**188**	**370**
Anthomyiidae	4	3	7
Atelestidae	0	15	15
Calliphoridae	2	1	3
Chloropidae	2	2	4
Culicidae	4	20	24
Dolichopodidae	6	1	7
Empididae	25	15	40
Fanniidae	27	9	36
Muscidae	50	76	126
Phoridae	1	2	3
Rhinophoridae	1	0	1
Scathophagidae	1	4	5
Simuliidae	1	1	2
Syrphidae	52	38	90
Tabanidae	1	0	1
Tachinidae	3	0	3
Tipulidae	2	1	3
**Hemiptera**	**0**	**3**	**3**
Hemiptera1	0	1	1
Hemiptera2	0	2	2
**Hymenoptera**	**62**	**67**	**129**
Andrenidae	2	4	6
Apidae	43	59	102
Ichneumonidae	2	1	3
Tenthredinidae	3	2	5
Hymenoptera1	0	1	1
Vespidae	12	0	12
**Lepidoptera**	**9**	**15**	**24**
Gracillariidae	3	6	9
Lycaenidae	0	1	1
Noctuidae	1	0	1
Nymphalidae	2	5	7
Papilionidae	1	0	1
Pieridae	2	2	4
Lepidoptera1	0	1	1
**Trichoptera**	**1**	**1**	**2**
Limnephilidae	1	1	2
**Grand Total**	**255**	**283**	**538**

Supplemental Table 5. Summary of 2023 dynamic network indices. All data were collected at Toolik. Comm. = community type; Cumu. = cumulative; Week = week of the growing season; Plant = number of plant species; Insect = number of insect families; $Links_1$ = total number of links; C = Connectance (0-1); N = Nestedness (0-100); NODF = Nestedness based on Overlap and Decreasing Fill (0-100); H_2' = Network-level specialization (0-1); Q = Modularity (0-1); $Links_2$ = mean number of links per plant species-insect family. NA indicates plant-insect interactions were insufficient to compute network metrices.

**Comm.**	**Week**	**Plant**	**Insect**	**$Links_1$**	**C**	**N**	**NODF**	**H2'**	**Q**	**$Links_2$**
Cumu.	1	1	1	1	1.00	NA	NA	NA	NA	0.50
Cumu.	2	4	8	12	0.38	17.32	48.53	0.38	0.33	1.00
Cumu.	3	6	10	20	0.33	14.44	55.00	0.36	0.34	1.25
Cumu.	4	10	13	30	0.23	17.99	39.57	0.49	0.40	1.30
Cumu.	5	9	15	30	0.22	15.27	44.38	0.44	0.37	1.25
Cumu.	6	14	16	33	0.15	14.14	24.72	0.60	0.53	1.10
Cumu.	7	10	10	22	0.22	14.52	43.15	0.48	0.49	1.10
Cumu.	8	6	14	23	0.27	19.90	42.61	0.54	0.46	1.15
Cumu.	9	4	5	8	0.40	29.02	37.50	0.62	0.44	0.89
Cumu.	10	0	0	0	NA	NA	NA	NA	NA	NA
Dry	1	0	0	0	NA	NA	NA	NA	NA	NA
Dry	2	3	3	4	0.44	24.21	33.33	0.78	0.33	0.67
Dry	3	5	9	16	0.36	15.07	56.70	0.40	0.36	1.14
Dry	4	9	13	27	0.23	17.70	41.45	0.50	0.41	1.23
Dry	5	5	13	24	0.37	19.25	51.23	0.43	0.33	1.33
Dry	6	8	12	20	0.21	28.85	24.36	0.69	0.53	1.00
Dry	7	7	6	12	0.29	22.90	34.26	0.60	0.48	0.92
Dry	8	4	9	15	0.42	25.71	45.79	0.49	0.40	1.15
Dry	9	3	3	5	0.56	37.08	33.33	0.56	0.31	0.83
Dry	10	0	0	0	NA	NA	NA	NA	NA	NA
Moist	1	0	0	0	NA	NA	NA	NA	NA	NA
Moist	2	1	1	1	1.00	NA	NA	NA	NA	0.50
Moist	3	2	2	3	0.75	0.00	100.00	0.68	0.25	0.75
Moist	4	3	3	5	0.56	27.08	33.33	0.46	0.33	0.83
Moist	5	5	5	9	0.36	22.98	45.00	0.57	0.34	0.90
Moist	6	10	10	19	0.19	23.73	22.13	0.70	0.59	0.90
Moist	7	7	8	14	0.25	22.25	34.01	0.63	0.51	0.93
Moist	8	5	11	14	0.25	27.93	24.36	0.77	0.54	0.88
Moist	9	2	4	4	0.50	46.84	0.00	1.00	0.38	0.67
Moist	10	0	0	NA	NA	NA	NA	NA	NA	NA
