# Supplementary material for: Spatio-temporal patterns in floral resources and plant-pollinator network structure in the Alaskan Arctic
Source: Front Plant Sci. 2025 Sep 24;16:1552422. doi: 10.3389/fpls.2025.1552422 (PMC12504194; doi:10.3389/fpls.2025.1552422)

Supplemental Figure 1. GAMM predictions (smooth trendlines) with 90% confidence bands (shaded) and daily means (points) for plot floral density (row 1) and proportion of plots in anthesis (row 2) for *Arctous alpina*. The boxplots show the temporal distribution of insect visits by Dipterans and Hymenopterans on *A. alpina*, including data from insect observations and collections (2022: 1 visit by Diptera, 35 visits by Hymenoptera, 0 Diptera collected, 8 Hymenoptera collected; 2023: 0 visits by Diptera, 26 visits by Hymenoptera, 0 Diptera collected, 6 Hymenoptera collected). Visitor observations and/or collections were done on days marked with a black tick. Grey vertical lines mark the beginning of each study week.

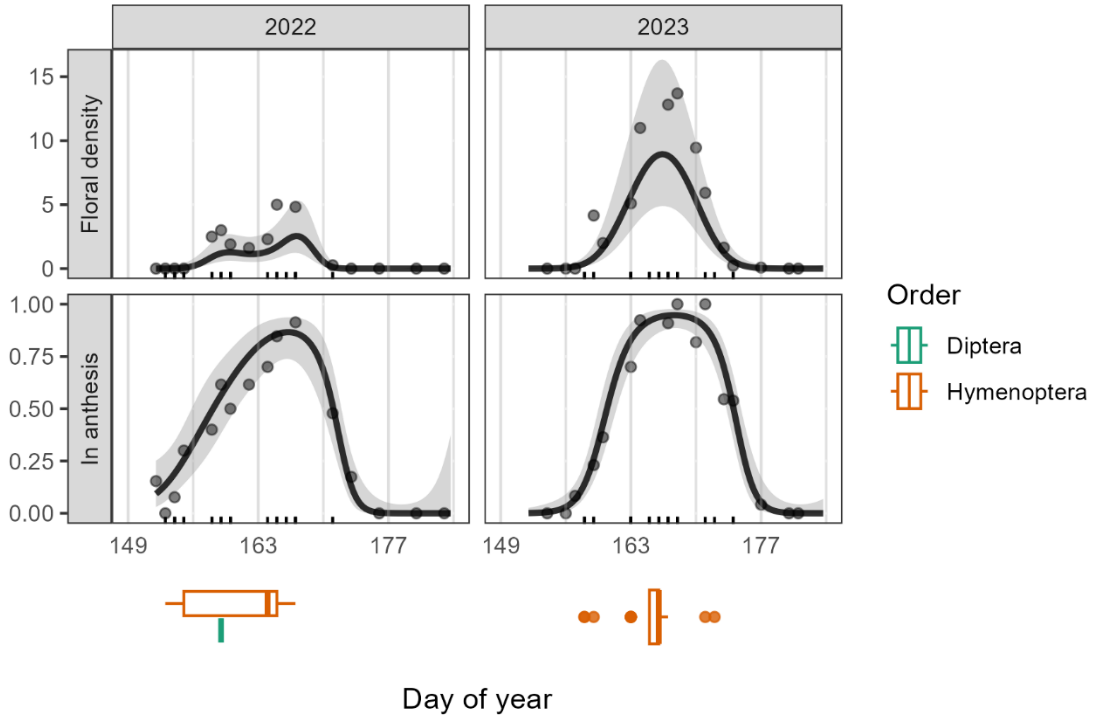

Supplemental Figure 2. GAMM predictions (smooth trendlines) with 90% confidence bands (shaded) and daily means (points) for plot floral density (row 1) and proportion of plots in anthesis (row 2) for *Dryas octopetala*. The boxplots show the temporal distribution of insect visits by Dipterans and Hymenopterans on *D. octopetala*, including data from insect observations and collections (2022: 15 visits by Diptera, 0 visits by Hymenoptera, 18 Diptera collected, 1 Hymenoptera collected; 2023: 5 visits by Diptera, 0 visits by Hymenoptera, 50 Diptera collected, 3 Hymenoptera collected). Visitor observations and/or collections were done on days marked with a black tick. Grey vertical lines mark the beginning of each study week (this plot starts on week 3).

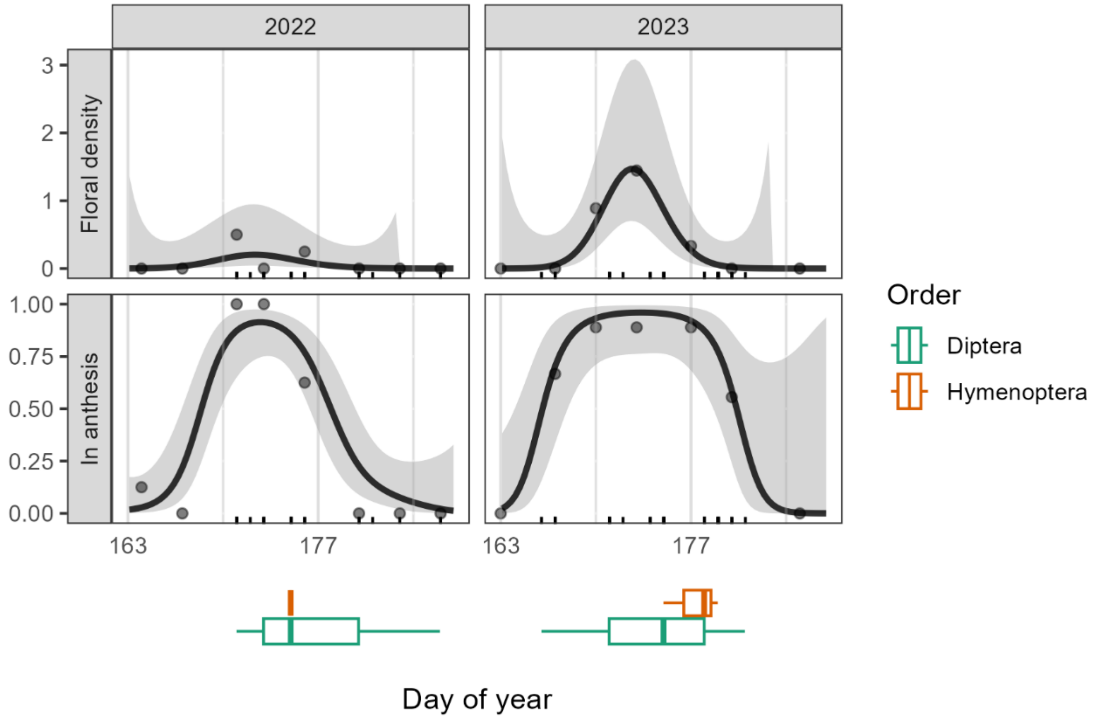

Supplemental Figure 3. GAMM predictions (smooth trendlines) with 90% confidence bands (shaded) and daily means (points) for plot floral density (row 1) and proportion of plots in anthesis (row 2) for *Kalmia procumbens*. The boxplots show the temporal distribution of insect visits by Dipterans and Hymenopterans on *K. procumbens*, including data from insect observations and collections (2022: 44 visits by Diptera, 240 visits by Hymenoptera, 6 Diptera collected, 9 Hymenoptera collected; 2023: 6 visits by Diptera, 12 visits by Hymenoptera, 6 Diptera collected, 13 Hymenoptera collected). Visitor observations and/or collections were done on days marked with a black tick. Grey vertical lines mark the beginning of each study week (this plot starts on week 2).

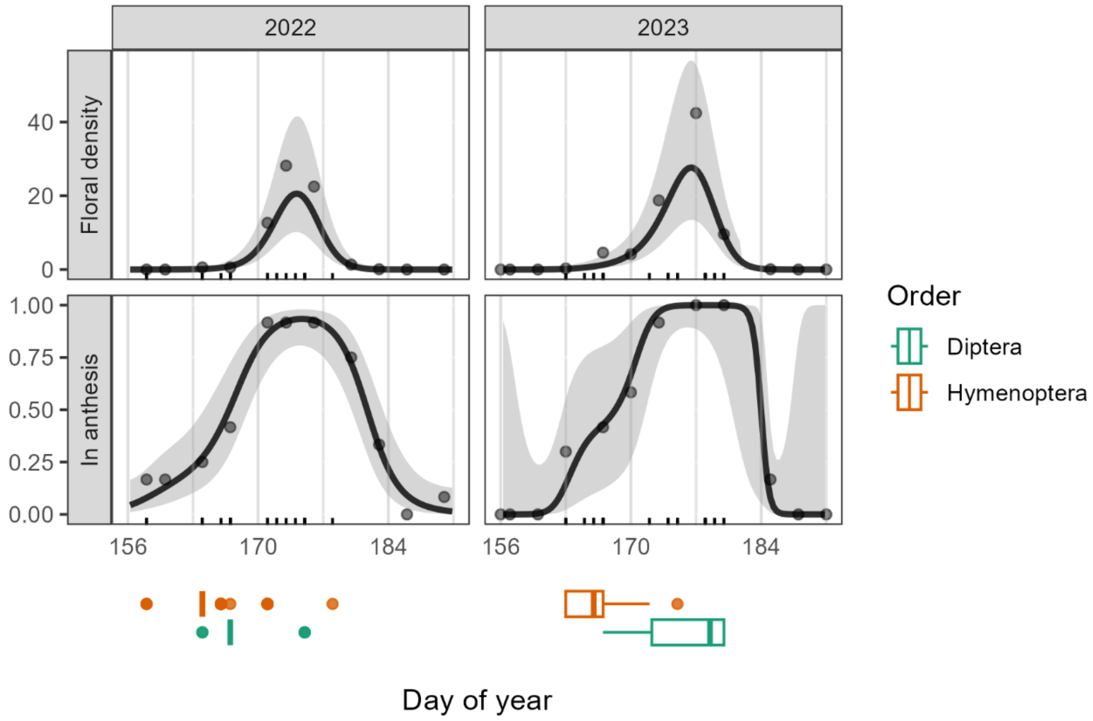

Supplemental Figure 4. GAMM predictions (smooth trendlines) with 90% confidence bands (shaded) and daily means (points) for plot floral density (row 1) and proportion of plots in anthesis (row 2) for *Rhododendron tomentosum*. The boxplots show the temporal distribution of insect visits by Dipterans on *R. tomentosum*, including data from insect observations and collections (2022: 13 visits by Diptera and 48 Diptera collected; 2023: 28 visits by Diptera and 29 Diptera collected). Hymenopterans made no visits and were not collected). Visitor observations and/or collections were done on days marked with a black tick. Grey vertical lines mark the beginning of each study week (this plot starts on week 4).

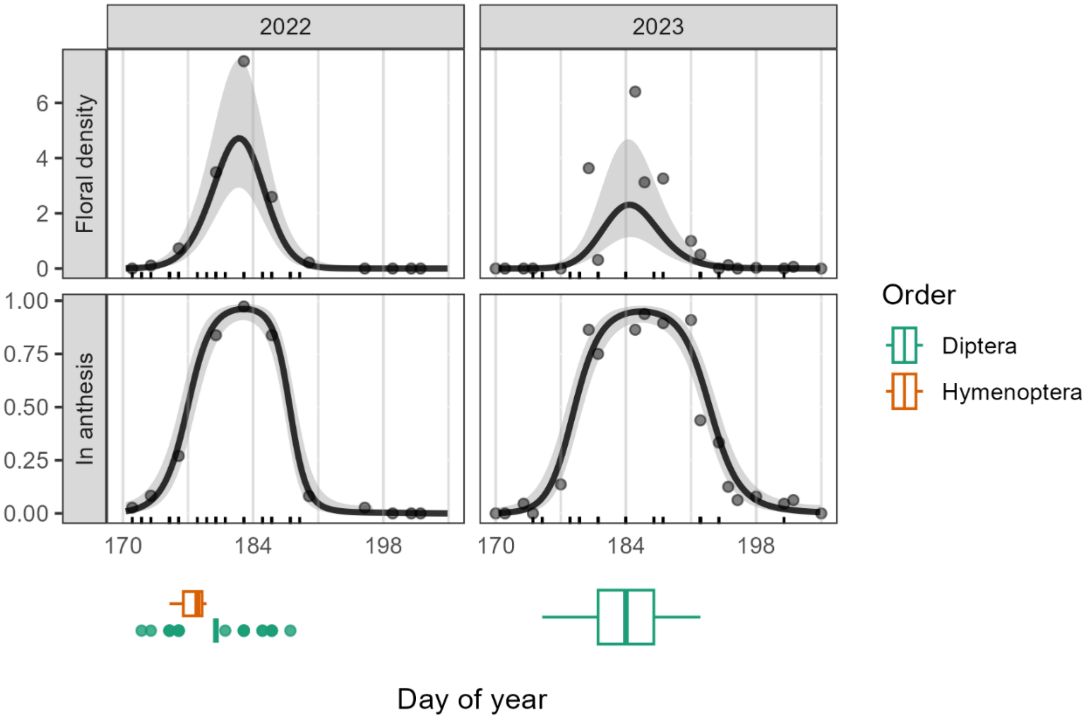

Supplemental Figure 5. GAMM predictions (smooth trendlines) with 90% confidence bands (shaded) and daily means (points) for plot floral density (row 1) and proportion of plots in anthesis (row 2) for *Vaccinium vitis-idaea*. The boxplots show the temporal distribution of insect visits by Dipterans and Hymenopterans on *V. vitis-idaea*, including data from insect observations and collections (2022: 4 visits by Diptera, 0 visits by Hymenoptera, 3 Diptera collected, 7 Hymenoptera collected; 2023: 0 visits by Diptera, 14 visits by Hymenoptera, 1 Diptera collected, 10 Hymenoptera collected). Visitor observations and/or collections were done on days marked with a black tick. Grey vertical lines mark the beginning of each study week (this plot starts on week 4).

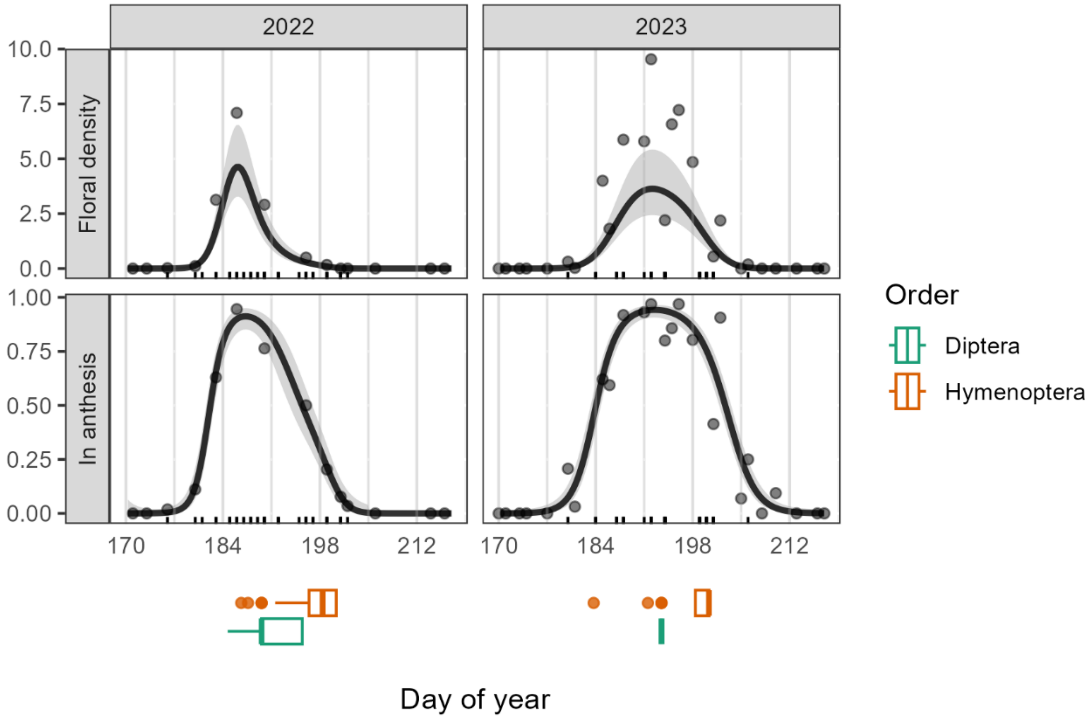

Supplemental Figure 6. Cumulative, static plant-insect visitation matrix based on visits observed during the 2022 growing season (at both Toolik and Imnavait sites). Plant species are represented on the y-axis and insect families are on the x-axis. A filled box indicates an observed link between a plant and insect. Each matrix is organized in a nested fashion such that the most generalized species/families occur at the top left and interact with the most other species/families.

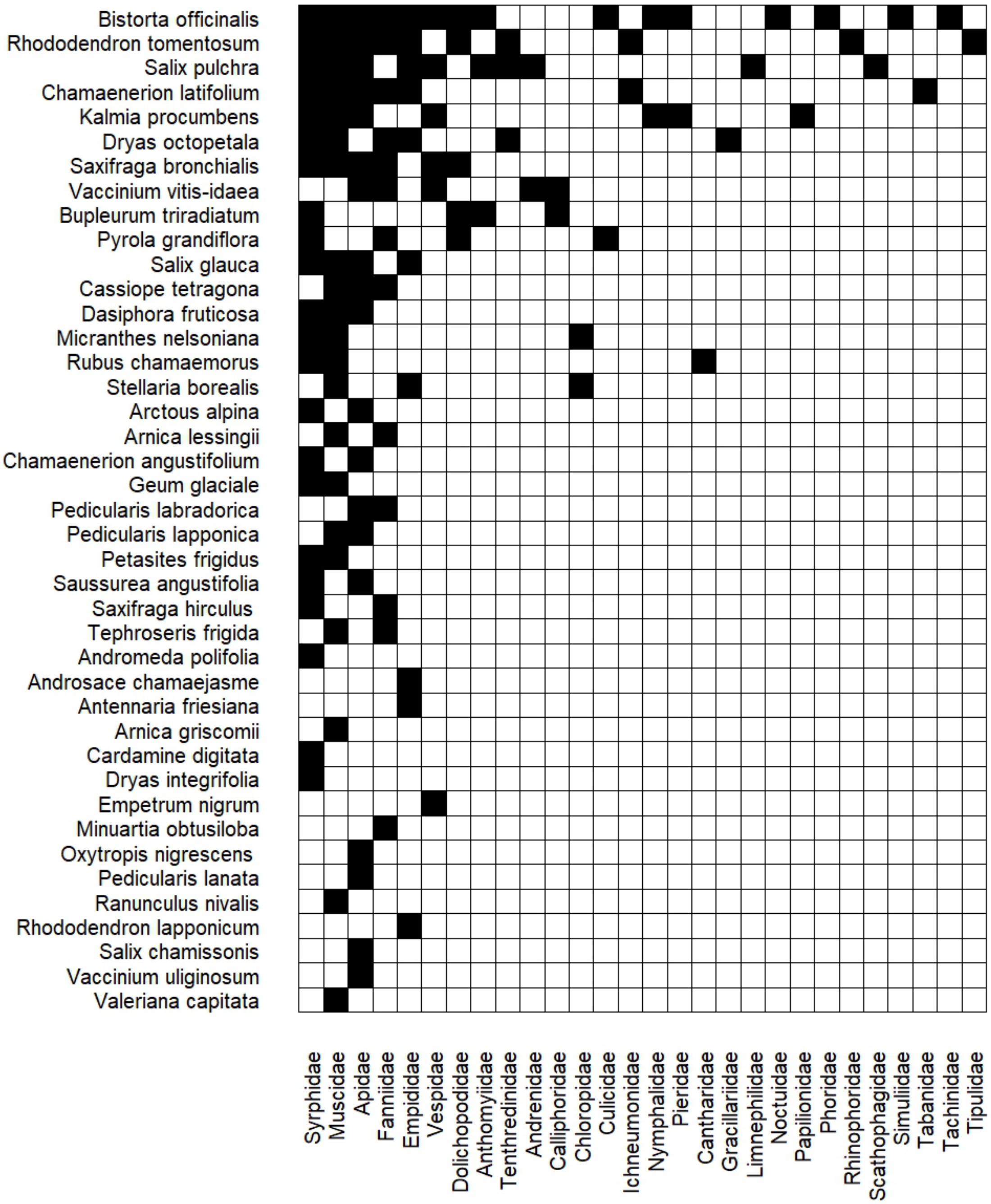

Supplemental Figure 7. Weekly sampling completeness by community type for 2023. Grey vertical lines mark the beginning of a study week. The y-axis shows sample completeness, calculated as the ratio of observed to estimated (Chao1) links, plants, and floral visitors.

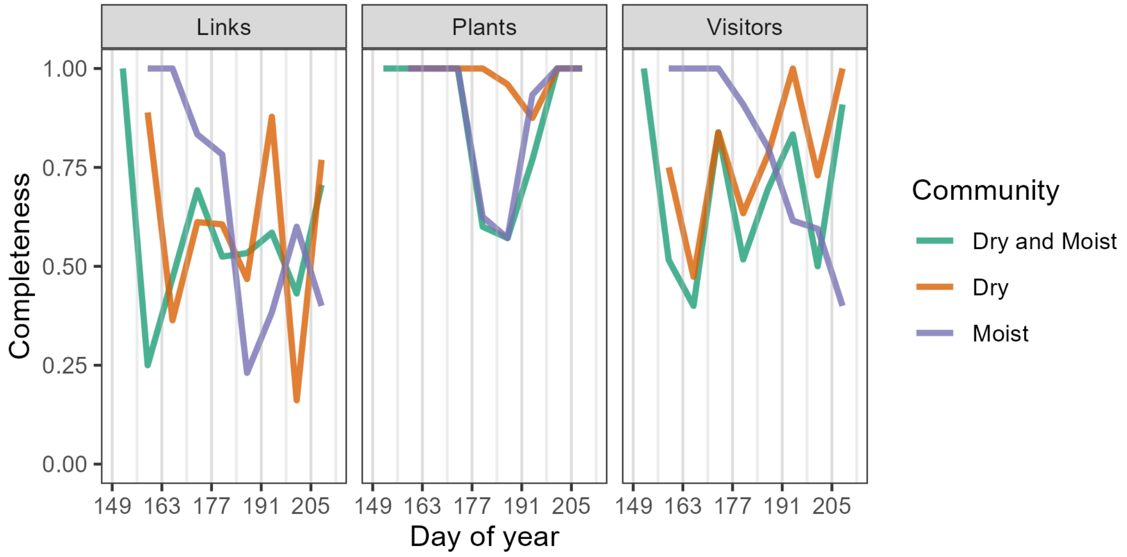

Supplemental Figure 8. Weekly sampling coverage by community type for 2023. Grey vertical lines mark the beginning of a study week. The y-axis shows sample coverage, representing the proportion of total links, plants, and floral visitors captured by the sample

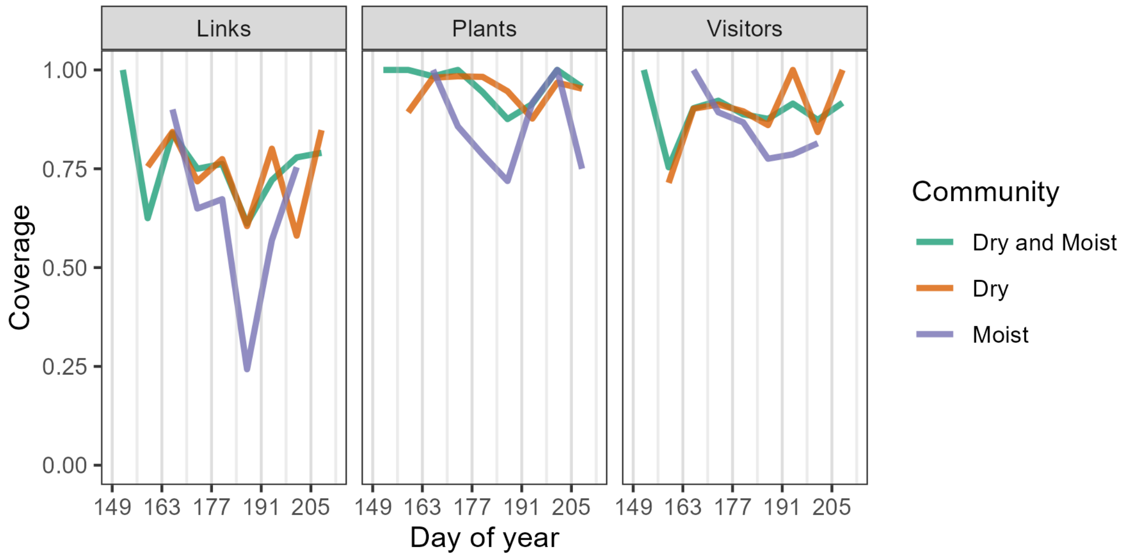

Supplement: Supplementary file 2 [file DataSheet2.pdf]
